# Tailoring Biopesticides: Amphiphile-Assisted Nanoprecipitation of Azadirachtin within a Glycine Matrix for Sustainable Agriculture, Enhanced Stability, and Larvicidal Efficacy against Fall Armyworm

**DOI:** 10.1021/acsomega.5c04028

**Published:** 2025-08-19

**Authors:** Michael Bae, Amanda Lewis, Shuhao Liu, Yashwanth Arcot, Yu-Ting Lin, Laxmi S. Viswanadha, Julio S. Bernal, Mustafa Akbulut, Luis Cisneros-Zevallos

**Affiliations:** † Artie McFerrin Department of Chemical Engineering, 14736 Texas A&M University, College Station, Texas 77843, United States; ‡ Department of Horticultural Science, Texas A&M University, College Station, Texas 77843, United States; § Jasper Department of Chemical Engineering, 578801 The University of Texas at Tyler, Tyler, Texas 75799, United States; ∥ J. Mike Walker ‘66 Department of Mechanical Engineering, Texas A&M University, College Station, Texas 77843, United States; ⊥ Department of Entomology, Texas A&M University, College Station, Texas 77843, United States; # Department of Materials Science and Engineering, Texas A&M University, College Station, Texas 77843, United States

## Abstract

The limited water solubility and environmental instability of natural pesticidal compounds impede their broader agricultural use. This study reports an amphiphile-assisted nanoprecipitation method to imbibe azadirachtin-rich neem seed extract (NSE) within a glycine carrier matrix, yielding a stable nanocomposite biopesticide. The formulation, prepared using polyoxyethylene sorbitan monooleate as a stabilizer and glycine as the matrix former, followed by lyophilization, exhibited a hydrodynamic diameter of ∼8 nm when redispersed in water. This glycine nanopesticide (GNP) significantly improved the photostability of azadirachtin under UV-AB irradiation (2000 μW/cm^2^); spectrophotometric analysis revealed a 27.7% reduction in photodegradation over a 4 day period compared to unformulated NSE powder demonstrated dialysis-based in vitro release assay showed sustained release, with 68.2 ± 2.1% released over 7 days, fitting an exponential model with a time constant of 37.6 h. Contact bioassays against *Spodoptera frugiperda* larvae revealed enhanced larvicidal potency. LC_50_ values showed a 1.5- to 6.6-fold improvement compared to unformulated NSE over 11 days. On day 7, GNP had an LC_50_ of 0.13 mg/mL, compared to 0.86 mg/mL for NSE powder. The nanoformulation also improved wettability on tomato leaves, reducing the contact angle from 99.0° ± 1.6° (DI water) to ∼60° at a concentration of 100 mg/mL GNP. This approach offers a practical method for improving the stability, delivery, and efficacy of hydrophobic biopesticides.

## Graphical abstract

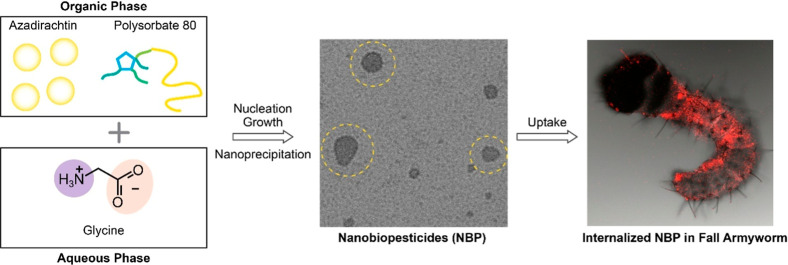

## 1 Introduction

Addressing the environmental challenges posed by synthetic agrochemicals requires exploring sustainable solutions.^1^ Among these, biopesticides derived from plant sources are recognized for their significant potential.^2,3^ Extracts obtained from *Azadirachta indica* (neem), enriched in the complex tetranortriterpenoid azadirachtin, exhibit potent insecticidal activity against a broad spectrum of pests, including the economically damaging *Spodoptera frugiperda*.^4–6^ The primary active compound, azadirachtin, is a nonpolar limonoid containing functional groups such as enol ethers, carboxylic esters, and epoxides.^7^ These molecular characteristics enable azadirachtin to disrupt insect physiology and behavior, ultimately leading to enhanced pest mortality and reduced fertility.^8^ These extracts also possess favorable toxicological profiles relative to nontarget species.^9^

However, translating the inherent bioactivity of neem compounds into efficacious pesticide applications encounters some obstacles rooted in the physicochemical characteristics of the active constituents. The predominantly hydrophobic molecular architecture of azadirachtin dictates poor aqueous solubility, thereby complicating formulation and delivery.^10^ Furthermore, the compound exhibits pronounced susceptibility to degradative processes, particularly hydrolytic cleavage^11^ and photolysis^12^ induced by environmental UV radiation, which inhibits persistence and overall bioefficacy.

Conventional oil-in-water macro- or microemulsions of NSE improve initial dispersibility but remain thermodynamically unstable.^13^ Gravitational creaming, coalescence, and limited interfacial protection can lead to rapid hydrolytic and photolytic loss of azadirachtin. Nanoscale platforms address some of these shortcomings but introduce different trade-offs.^14,15^ Nanoemulsions, which are often produced by microfluidization or phase-inversion temperature cycling to shrink droplet diameters to <200 nm, can suppress creaming and enhance cuticular penetration.^16,17^ Nevertheless, the liquid core still promotes Brownian diffusion of biaoctive species, while Ostwald ripening (driven by the high Laplace pressure of ultrafine droplets) progressively broadens the size distribution and accelerates payload leakage.^18,19^ Polymeric nanocapsules prepared by solvent displacement, interfacial polymerization, or electrospraying provide a solid shell that better retards photolysis and hydrolysis.^20,21^ Yet, they typically require chlorinated or aromatic solvents and exhibit incomplete biodegradability timelines that may not align with cropping cycles.^22,23^ Solid-lipid nanoparticles and nanostructured lipid carriers use long-chain triglycerides or fatty acids that crystallize on cooling, generating matrices able to entrap NSE with 55–80% efficiency.^15,24^ Across these systems, the initial burst release often exceeds 40–60% of the payload, struggling to provide the multiday residual activity required for pests such as *S. frugiperda*.^25^ Importantly, most of these platforms do not produce sub-20 nm particles, which is the size range regarded as essential for systemic delivery through stem apoplastic channels and sieve-plate pores.^26–28^ In sum, it is still a challenge for existing formulations to achieve the combined targets of (i) high azadirachtin loading, (ii) ultrasmall size, (iii) robust photochemical shielding, (iv) predictably sustained release, and (v) scalable, solvent-lean manufacturing using food-grade excipients, which are gaps that motivate the amphiphile-assisted glycine nanoprecipitation strategy advanced in this work.

This study employs amphiphile-mediated nanoprecipitation to embed NSE bioactives within a glycine matrix, followed by lyophilization to yield a solid nanocomposite. Briefly, NSE and an amphiphilic stabilizer (polyoxyethylene sorbitan monooleate) dissolved in isopropyl alcohol are rapidly injected into an aqueous glycine solution. This sudden solvent change is intended to induce NSE phase separation due to poor miscibility (high positive Δ*G*_mix_), leading to supersaturation. The resulting rapid nucleation and growth, potentially limited by the fast precipitation kinetics, yield nanoparticles typically below 10 nm. The cosolubilized amphiphile is thermodynamically forced to adsorb onto the nascent NSE nanoparticles, providing steric stabilization that inhibits significant aggregation and Ostwald ripening. During subsequent lyophilization, the glycine solidify into a continuous matrix, to immobilize the nanoparticles, prevent fusion, and serve as a cryo-protectant.

The novelty inherent to this methodology arises from the synergistic effect of rapid nanoprecipitation kinetics, modulated by the interfacial adsorption of an amphiphilic surfactant during solvent elimination by lyophilization, and the in situ generation of a functional and biodegradable glycine matrix. This approach differs from (a) conventional emulsification, which necessitates stabilization of preformed liquid phases, and (b) typical polymeric nanoencapsulation techniques often reliant on polymer deposition or interfacial polymerization phenomena. Our strategy seeks to exploit the unique attributes of zwitterionic glycine, not merely as an inert excipient, but as a matrix-forming component that participates in the in situ architectural stabilization of nanoparticles during formulation. This offers potential advantages regarding process simplicity, utilization of biodegradable constituents. Furthermore, the solid glycine matrix provides enhanced photostability through physical shielding, allows for tunable release kinetics governed by matrix rehydration, dissolution, and diffusion processes, and facilitates achieving the sub-20 nm particle size range regarded as essential for systemic delivery in plants. This approach not only improves bioefficacy but also contributes to more environmentally benign pest management solutions. Such advances are particularly significant in light of increasing regulatory and ecological pressures to reduce synthetic pesticide usage.

Consequently, the objectives guiding this research are (i) synthesis of this glycine nanopesticide and comprehensive characterization of the resultant solid nanocomposites and the properties upon aqueous redispersion, involving particle size distribution (via DLS) and morphology (via TEM); (ii) quantitative evaluation of the enhancement in photostability conferred by the glycine matrix through quantitative monitoring of azadirachtin degradation under defined UV stress conditions; (iii) elucidation of the in vitro release kinetics governing azadirachtin liberation from the solid dispersion into an aqueous sink; and (iv) supporting assessment of the biological consequences of this formulation strategy through determination of larvicidal efficacy (LC_50_, mortality kinetics, growth inhibition) against *S. frugiperda*. Here, the fall armyworm (*S. frugiperda*), a highly invasive lepidopteran pest, was focused on because it has become a critical global threat to agriculture, causing substantial damage to over 350 economically important crop species (particularly maize, rice, sorghum, and cotton—across Africa, Asia, and the Americas).^29,30^

## 2 Materials and Methods

### 2.1 Materials

High-purity glycine (>99.5%, cell culture reagent, Alfa Aesar, Haverhill, MA, USA, CAS# 56-40-6) was utilized as the primary matrix-forming component for the nanoprecipitation process and subsequent composite formation. Neem seed extract (NSE; Fortune Aza Technical, Fortune Biotech Ltd., Andhra Pradesh, India), provided as a powder, contained 21% w/w azadirachtin and 24% w/w other limonoids, with the balance comprising polyphenolics, lignins, fatty acid esters, and lipids, according to the specifications of the manufacturer. Polyoxyethylene sorbitan monooleate (Tween 80, TW; TCI America, Portland, OR, USA, CAS# 9005-65-6) served as the amphiphilic stabilizer during the nanoprecipitation of NSE components. Isopropyl alcohol (IPA; 99%, VWR International, Radnor, PA, USA, CAS# 67-63-0) was used as the water-miscible organic solvent to dissolve both the NSE and the amphiphilic stabilizer, forming the organic phase prior to rapid injection into the aqueous phase for nanoprecipitation. Nile red fluorescent dye (98%, TCI America, Portland, OR, USA, CAS# 7385-67-3) was used for the confocal microscopy studies in fluorescence tagging. Uranyl acetate (SPI-CHEM, West Chester, PA, USA) was used for TEM staining. All aqueous solutions were prepared using deionized (DI) water (≥18 MΩ·cm resistivity, Milli-Q system, MilliporeSigma, Burlington, MA, USA). Tomato seeds (Cultivar Red Robin) were obtained from Eden Brothers (Arden, NC, USA). *S. frugiperda* eggs and artificial diet were sourced from Benzon Research (Carlisle, PA, USA).

### 2.2 Preparation of Glycine Nanopesticide (GNP)

This process utilizes an amphiphile-assisted nanoprecipitation method followed by lyophilization. First, an aqueous glycine (GLY) phase was prepared by dissolving 0.45 g of GLY powder in 100 mL of DI water, assisted by brief bath sonication (Branson 2800, Branson Ultrasonics Corporation, Danbury, CT, USA) for 10 min at room temperature (∼22 °C) to ensure complete dissolution. Concurrently, an organic phase containing the active ingredient and stabilizer was prepared by dissolving 0.05 g of NSE powder and 0.50 g of TW (corresponding to a 10:1 w/w ratio of TW/NSE) in 10 mL of IPA. This mixture was also sonicated for 10 min at ∼22 °C to ensure homogeneity. The organic phase (10 mL) was then rapidly injected into the aqueous GLY phase (100 mL) over approximately 1 min while the aqueous phase was under continuous bath sonication. Sonication was continued for an additional 10 min postinjection to facilitate stabilization of the resulting nanoparticle dispersion. This procedure yielded the “as-prepared” GNP nanosuspension.

### 2.3 Freeze-Drying of GNP Nanosuspension

The as-prepared GNP nanosuspension (containing ∼110 mL of water/IPA 10:1 v/v mixture) was rapidly frozen by immersing the container (e.g., 500 mL round-bottom flask) in liquid nitrogen while swirling until solidified. The frozen sample was then subjected to lyophilization using a benchtop freeze-dryer (FreeZone 4.5 L −105 °C Cascade Console Freeze-Dry System, model 7750040, Labconco Corporation, MO, USA). The process was run with a condenser temperature below −50 °C and a vacuum pressure below 0.2 mbar (typically 0.02–0.05 mbar) for 48 h to ensure complete sublimation of water and IPA. The resulting product, termed glycine nanopesticide (GNP) powder, was a lightweight, porous, foam-like solid cake. The powder was carefully collected, transferred to sealed glass vials, and stored with desiccant at 4 °C until further use.

### 2.4 Characterization of GNP Nanosuspension and Lyophilized Powder

The hydrodynamic diameter and polydispersity index (PDI) of the as-prepared GNP nanosuspension, as well as the redispersed lyophilized powder were measured using dynamic light scattering (DLS). Measurements were performed at 25 °C using a Zetasizer Nano ZS (Malvern Panalytical Ltd., Malvern, UK) equipped with a He–Ne laser (633 nm). Samples were diluted appropriately with DI water prior to measurement to achieve optimal scattering intensity (typically targeting a count rate between 100 and 500 kcps). Zeta potential measurements were conducted using the same instrument, employing Laser Doppler Velocimetry and Phase Analysis Light Scattering (M3-PALS technique) in folded capillary cells (DTS1070, Malvern Panalytical). All DLS and zeta potential measurements were performed in triplicate.

Transmission electron microscopy (TEM) was used to visualize nanoparticle morphology and size. A small droplet (∼5 μL) of the diluted GNP nanosuspension (typically 0.1 mg/mL total solids, both as-prepared and redispersed lyophilized powder) was deposited onto a 300-mesh carbon-coated copper grid (Ted Pella, Inc., Redding, CA, USA). After 1 min, excess liquid was wicked away using filter paper. Negative staining was performed by applying a droplet of 2% (w/v) aqueous uranyl acetate solution for 1 min, followed by wicking away the excess stain. Grids were allowed to air-dry completely before imaging. TEM imaging was conducted using a FEI Tecnai G2 F20 X-TWIN TEM (FEI Company, part of Thermo Fisher Scientific, Hillsboro, OR, USA) operated at an accelerating voltage of 200 kV.

### 2.5 Release Kinetics of Azadirachtin from GNP

To study the release kinetics of the nanoformulation, we relied on a dialysis membrane-based approach as described elsewhere.^31–33^ A precisely weighed amount of lyophilized GNP powder corresponding to the solids obtained from the initial 110 mL preparation, i.e., ∼0.505 g containing 0.45 g GLY, 0.050 g TW, and 0.005 g NSE was reconstituted in 11 mL of a water/IPA (10:1 v/v) mixture, forming a concentrated suspension. This suspension was transferred into a presoaked dialysis bag (Spectra/Por 3, MWCO 3.5 kDa, Standard grade RC Tubing, Fisher Scientific, Waltham, MA, USA, Cat# S132725) and securely sealed with dialysis clamps (Spectra/Por Closures, Fisher Scientific). The sealed bag was immersed in 539 mL of the same water/IPA (10:1 v/v) mixture (release medium) in a covered 1 L beaker, representing a 49-fold volume excess relative to the internal volume (total volume 550 mL, achieving sink conditions), maintained at 22 °C with constant gentle stirring (100 rpm magnetic stirrer). At predetermined time intervals (∼0, 1, 2, 4, 8, 12, 24, 48, 72, 96, 120, 144, and 168 h), 3 mL aliquots were withdrawn from the external release medium. The absorbance spectrum (200–800 nm) of each aliquot was measured immediately using a UV/vis spectrophotometer (Shimadzu UV-1800, Shimadzu Scientific Instruments, Kyoto, Japan) in a 1 cm path length quartz cuvette. The concentration of released components was determined based on the absorbance peak characteristic of azadirachtin (∼220 nm). After each sampling, 3 mL of fresh release medium was immediately added back to the beaker to maintain constant volume and sink conditions. Control experiments were conducted concurrently using (i) the carrier matrix alone (reconstituted powder containing 0.45 g GLY + 0.050 g TW) and (ii) bulk NSE powder (0.005 g dispersed in 11 mL water/IPA) subjected to the same dialysis procedure. The absorbance spectrum of the carrier matrix control was subtracted from the GNP sample spectrum at each time point to isolate the signal attributed specifically to NSE components. Standard calibration curves for GNP, carrier matrix, and NSE in the release medium were generated prior to the experiment using six known concentrations (*R* ^2^ > 0.99 for all curves). The entire release study was performed in triplicate. The release profiles of azadirachtin were fitted using a single-exponential decay model in OriginPro2018 software (OriginLab Corporation, Northampton, MA, USA). This model confirms a good fit (*R* ^2^ > 0.98), supporting the characterization of the release kinetics as approximately first-order up to 168 h.

### 2.6 Photostability Evaluation under UV Irradiation

The photostability of the glycine nanopesticide (GNP) powder was evaluated under simulated sunlight conditions using a UV-AB lamp (160 W, REPTI ZOO, Miami, FL, USA). The experimental setup maintained a 15 cm distance between the light source and the sample. The incident flux was calibrated to ∼2000 μW/cm^2^ (1850 ± 306 μW/cm^2^), as measured by a digital UV-AB light meter (UV513AB, General Tools, Secaucus, NJ, USA; spectrum range: 290–370 nm, calibration point: 365 nm).

Aqueous suspensions of GNP powder and carrier powder were prepared separately and deposited in triplicate drops on quartz blocks. Concurrently, neem seed extract (NSE) powder was dissolved in isopropyl alcohol and applied similarly. The coated quartz blocks were desiccated overnight in a biological safety cabinet prior to analysis.

Photodegradation kinetics were monitored via UV/vis spectroscopy, tracking the decay of absorbance intensity at 220 nm as a function of UV exposure time. Control samples, shielded from light irradiation, were maintained at room temperature to account for potential thermal degradation effects. The photodegradation assay was replicated 3–5 times to ensure statistical robustness.

### 2.7 *S. frugiperda* Rearing and Preparation

*S. frugiperda* eggs (Benzon Research) were received on paper sections and incubated inside sealed plastic bags with moist paper towels at room temperature (∼22–24 °C). Upon hatching (typically 3–4 days), sections of paper with newly hatched neonates were placed in ventilated plastic containers (e.g., 16 oz deli cups) containing ∼20 cm^3^ blocks of artificial insect diet (Benzon Research; soy flour/wheat germ base). Containers were maintained under a 12:12 h light/dark cycle using fluorescent lights at ambient room temperature (∼22–24 °C). Larvae were allowed to develop on the diet. First instar larvae (within 24 h of hatching, designated day 1 posthatching) were utilized for all bioassays and imaging unless otherwise specified. This early larval stage is frequently employed in initial efficacy screening studies reported in the literature due to its high sensitivity and relatively rapid development, facilitating accelerated comparative assessments.^34–36^ While acknowledging that field efficacy involves various instars, the use of first instars here primarily serves to provide supporting evidence for enhanced biological activity resulting from the nanoformulation process.

### 2.8 Larval Imaging (SEM and Confocal Microscopy)

For scanning electron microscopy (SEM), larvae (untreated controls on day 1 and day 3; GNP-treated on day 3) were first immobilized by chilling at 4 °C for approximately 30 min, followed by fixation (e.g., in 2.5% glutaraldehyde in phosphate buffer, followed by dehydration through an ethanol series) by direct immobilization via refrigeration (4 °C for 24 h). Immobilized/dried larvae were mounted on aluminum stubs using double-sided carbon tape and sputter-coated with a thin layer (∼10 nm) of gold–palladium (208 HR Sputter Coater, Cressington Scientific Instruments, Watford, UK) to prevent charging. Imaging was performed using a field emission SEM (JSM-7500F; JEOL USA, Peabody, MA, USA) at accelerating voltages of 5–10 kV. Measurements of mandible and spiracle dimensions were performed using the integrated SEM analysis software.

For confocal microscopy to assess potential GNP uptake, Nile red fluorescent dye was incorporated into the GNP formulation during the organic phase preparation at 1% w/w relative to NSE content (0.0005 g Nile red per 0.05 g NSE). Day 1 larvae were briefly immersed (2 s) in an aqueous suspension of this Nile red-tagged GNP (100 mg/mL). Treated larvae were transferred to individual wells of a bioassay tray containing a 1 cm^3^ diet block and allowed to feed/rest for 1 h. Larvae were then immobilized by chilling at 4 °C for 24 h. Whole larvae were mounted on glass slides in a suitable mounting medium (e.g., glycerol or specialized mounting medium). Imaging was performed using a ZEISS LSM 780 NLO Multiphoton Microscope (Carl Zeiss Microscopy GmbH, Jena, Germany) using a 561 nm laser line for Nile red excitation and detecting emission between 570 and 650 nm. Z-stack images were acquired through the larval body. Control groups included larvae treated with (i) a 1:100 dilution of the tagged-GNP suspension (1 mg/mL GNP, 0.5 μg/mL Nile red), (ii) Nile red alone dispersed in water (50 μg/mL), and (iii) DI water only. Laser power and detector gain settings were kept constant across all samples for valid comparison of fluorescence intensity.

### 2.9 Mortality and Growth Bioassays

A contact immersion bioassay protocol was employed. Aqueous suspensions of GNP powder were prepared fresh daily by reconstituting the lyophilized powder in DI water to achieve six final concentrations: 1000, 100, 10, 1, 0.1, 0.01 mg/mL, plus a 0 mg/mL DI water control. For comparison, aqueous dispersions of bulk NSE powder were prepared at four concentrations (50, 5, 0.5, 0.05 mg/mL) and solutions of the carrier components alone (GLY + TW, maintaining the 9:10 GLY/TW weight ratio from the formulation) were prepared at three concentrations (950, 95, 9.5 mg/mL). The carrier concentrations approximately match the amount present in the 1000, 100, and 10 mg/mL GNP suspensions, respectively. NSE concentrations were chosen based on preliminary trials.

Day 1 *S. frugiperda* larvae were used. Using soft forceps, individual larvae were gently picked up and fully immersed for exactly 2 s in the designated test suspension/solution. After immersion, excess liquid was briefly drained by touching the larva to filter paper, and the larva was placed into an individual well of a 24-well polystyrene bioassay tray containing a small piece (∼0.5 cm^3^) of artificial diet. Thirty larvae were tested per concentration, allocated across three replicate trays (10 larvae per tray). Trays were covered with ventilated plastic lids (e.g., Thermo Scientific Nunc Edge 2.0 Plates) and maintained under controlled conditions (12:12 h L/D photoperiod, ∼24 °C).

Mortality was assessed daily for 11 days. Larvae were recorded as dead if they exhibited no movement after being gently prodded with a fine probe for 5 s. Larval length was measured daily by photographing each tray with an overhead camera including a reference scale. Lengths were measured from the digital images using ImageJ software (Version 1.53+, National Institutes of Health, Bethesda, MD, USA). Percent mortality was calculated for each replicate. Average mortality and length (±standard error) were calculated across replicates for each treatment and time point.

Median lethal concentration (LC_50_) values were interpolated for each day using a four-parameter sigmoidal dose–response model implemented via the AAT Bioquest LC_50_ Calculator (https://www.aatbio.com/tools/lc50-calculator). This tool estimates LC_50_ values based on curve fitting of mortality data across concentrations. Statistical analyses (two-way ANOVA with time and concentration as factors, followed by Tukey’s HSD posthoc test for pairwise comparisons) were performed using JMP software (Version 16.1.0, JMP Statistical Discovery LLC, Cary, NC, USA) to evaluate treatment effects on mortality and growth inhibition. Statistical significance was defined at *p* < 0.05.

### 2.10 Leaf-Feeding Bioassay

Fresh, undamaged leaflets were detached from 5 week-old Red Robin tomato plants. Leaflets were briefly dipped (adaxial and abaxial surfaces) into the same GNP aqueous suspensions prepared for the contact assay (100, 10, 1, 0.1 mg/mL, and DI water control) and allowed to air-dry for ∼30 min. First instar larvae (day 1) were starved for 2 h. Individual starved larvae were placed in bioassay tray wells, each containing one treated tomato leaflet as the sole food source. After 24 h of exposure to the treated leaflet, the leaflet was removed, and a standard 1 cm^3^ block of artificial diet was provided to prevent mortality from starvation unrelated to treatment effects. Daily mortality assessments and data analysis (LC_50_ calculation, statistical comparisons to contact assay results) were conducted for 11 days following the same procedures outlined in the section above.

### 2.11 Plant Growth and Foliar Wetting Analysis

Red Robin tomato plants *(Solanum lycopersicum* var. cerasiforme) were grown from seed in 4-in. pots containing standard potting mix (Pro-Mix BX Mycorrhizae, Premier Tech Horticulture, PA, USA). Plants were cultivated in a research greenhouse section at Texas A&M University, utilizing standard potting mix and fertilization protocols. Greenhouse temperature and relative humidity were monitored but subject to typical fluctuations, generally targeted within ranges suitable for tomato growth (e.g., ∼20–25 °C day/15–20 °C night and 50–80% RH). Plants were watered as needed and fertilized weekly with a 20–20–20 NPK water-soluble fertilizer. Fully expanded, healthy leaves from ∼5 week-old plants were used for wetting analysis. Static contact angle measurements were performed on the adaxial (upper) surface of freshly detached leaves. Aqueous suspensions of GNP were prepared at five concentrations: 100, 10, 1, 0.1, 0.01 mg/mL, and a DI water control. Using a precision microliter syringe or pipet, droplets of exactly 5 μL volume were carefully deposited onto the leaf surface from a minimal height (∼1 cm). Digital images of the sessile droplets were captured within 10 s of deposition using an in-house goniometer system described elsewhere.^37–39^ The static contact angle, θ, was determined from the captured images using image analysis software employing the sessile drop method (e.g., ImageJ with LBADSA plugin). At least five measurements on different leaves were averaged for each concentration.

## 3 Results and Discussion

### 3.1 Size, Structural, and Colloidal Characteristics of Composite Biopesticide before and after Freeze-Drying

The size, structure, and zeta potential are fundamental parameters for characterizing nanoparticles and understanding their localization, distribution, and interactions in plants and pests upon application.^40^ Accordingly, we first present the dimensional and structural attributes of GNP nanoparticles. Figure 1 illustrates the particle size distribution from triplicate measurements (*n* = 3) and TEM micrographs of the as-prepared nanoformulation, as well as the aqueous redispersion of the lyophilized powder. The primary particle size was approximately 8 nm, as evidenced by the DLS peak observed in Figure 1a. TEM micrographs presented in Figure 1b,c offer valuable insights into the morphology of the nanopesticide particles in both the as-prepared state and after freeze-drying. The as-prepared nanopesticide shows a dispersion of roughly spherical nanoparticles. A large fraction of particles were below 10 nm while occasional larger particles were also noted in TEM. The TEM image of the nanopesticide after freeze-drying and aqueous redispersion shows a similar overall appearance and size-characteristics to the as-prepared sample. The particles maintain their roughly spherical shape and seem to be well-dispersed, suggesting that the freeze-drying process did not cause noticeable irreversible aggregation. Regarding the comparison of the size characteristics from two methods (i.e., DLS and TEM), it is important to note that DLS measures the hydrodynamic diameter in solution, which reflects the effective size of the particle diffusing in the solvent,^41^ including the core NSE material plus any solvated layers associated water molecules. Conversely, TEM visualizes the electron-dense core of the nanoparticles after they have been deposited and dried onto a grid with staining.^42^ This provides direct morphological information and allows for measurement of the primary particle dimensions, but sample preparation itself can induce effects such as aggregation upon drying/concentrating.^43^

**Figure 1:**
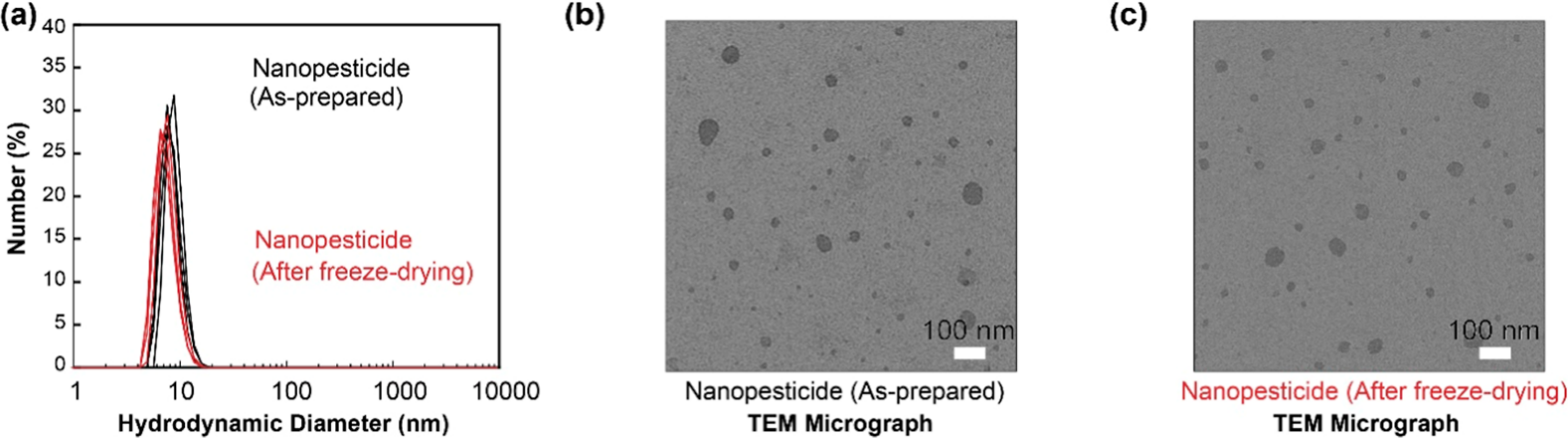
(a) The number-averaged particle size (hydrodynamic diameter) distribution of GNP (*n* = 3), and transmission electron microscope image of (b) as-prepared GNP and (c) resuspended GNP after lyophilization, confirming the preservation of morphology and absence of significant aggregation.

The particle sizes given above were for the optimum composition having a 10:1 emulsifier to neem seed extract ratio. We also studied the effect of composition on the nanopesticide size by varying emulsifier to neem seed extract ratios of 1:1, 2:1, 4:1, 6:1, 8:1, and 10:1, as depicted in Figure S1. Due to other extensive elements of the manuscript, we placed the formulation optimization details in the Supporting Information. The main observation was that as the amount of amphiphile increased from 4:1 to 10:1, a progressive decrease in the polydispersity index (PDI) from 0.39 ± 0.03 to 0.22 ± 0.02 was observed. This trend reflects enhanced adsorption of the amphiphile onto nascent nanoparticle surfaces, which lowers interfacial tension and provides steric stabilization. Increased surfactant concentrations improve surface coverage, thereby reducing coalescence and limiting size variation during growth. Notably, formulations with ratios below 4:1 failed to exhibit a clear peak in the vicinity of 10 nm, suggesting suboptimal nanoparticle formation under these conditions.

The zeta potentials were −5.8 ± 0.4 mV for the as-prepared nanoformulation and −10.5 ± 1.1 mV for the redispersed lyophilized powder, based on triplicate measurements (*n* = 3) (Figure 2). The relatively low magnitude of these zeta potentials indicates that electrostatic repulsion is not the primary mechanism driving the colloidal stability of the GNP nanoparticles. Instead, the observed stability against aggregation is primarily attributed to steric stabilization conferred by the nonionic amphiphile, polyoxyethylene sorbitan monooleate (TW). The long, hydrophilic polyoxyethylene chains of TW molecules adsorbed onto the nanoprecipitate surface extend into the aqueous phase, creating a physical, solvated barrier that hinders close approach and aggregation of the nanoparticles.^44,45^

**Figure 2:**
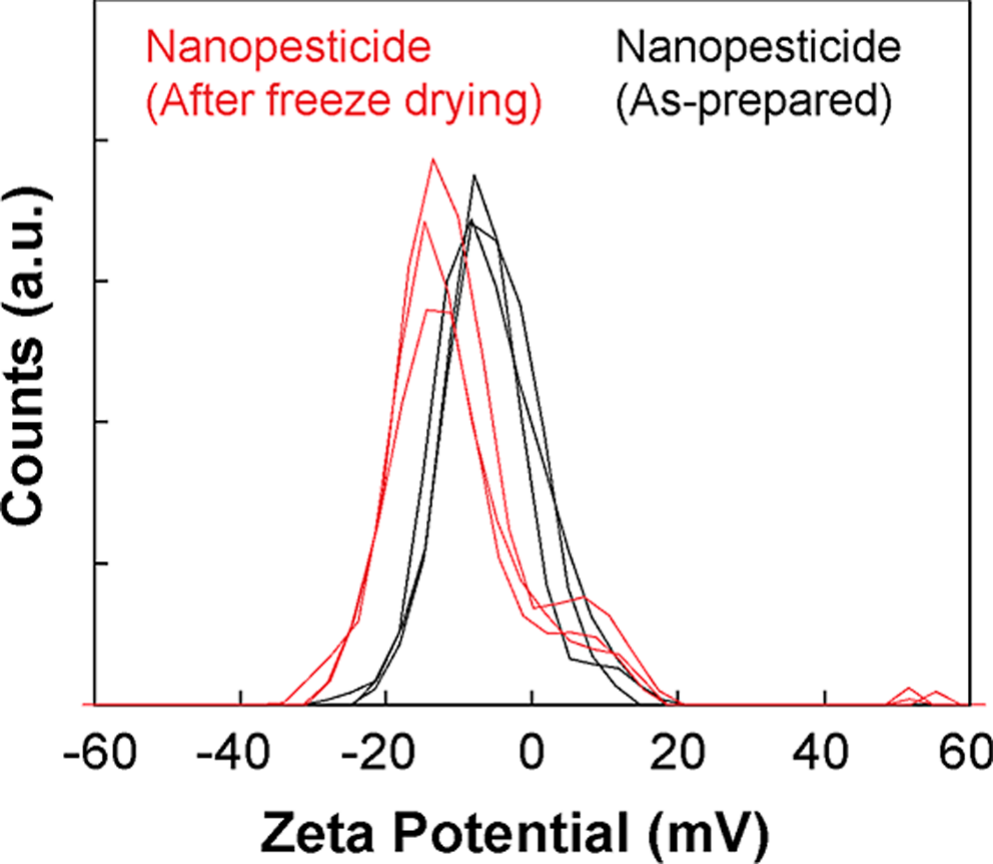
Zeta-potential distribution of glycine nanopesticide before and after freeze-drying (*n* = 3). The graph compares the zeta potential of the nanopesticide in its as-prepared state (black lines) to its state after freeze-drying and redispersion (red lines). The as-prepared nanopesticide shows a slightly narrower distribution centered closer to 0 mV, while the freeze-dried and redispersed nanopesticide exhibits a slightly broader distribution shifted toward more negative values.

Regarding the relative differences before and after lyophilization, in postlyophilization, the structural rearrangement of TW encapsulating the lipophilic constituents of NSE may induce capillary forces on the core, potentially intensifying the surface charge due to the exposure of ether, ester, and hydroxyl moieties from TW. In the absence of IPA in the lyophilized redispersion, two primary mechanisms appear to prevent aggregation and precipitation of the neem seed extract: (i) during the lyophilization process, the Laplace pressure induced by the self-assembly of amphiphilic blocks facilitates the sequestration of lipophilic compounds within the core of each nanoparticle^46,47^ and (ii) glycine molecules establish an extensive hydrogen bonding network in the aqueous medium surrounding each nanoparticle.^48,49^ This interparticle attractive force can immobilize in loosely bound state the nanoparticles and inhibits collision-induced aggregation. GNP demonstrated high aqueous dispersibility, attributed to the electronegative and hydrophilic characteristics of the outer layer formed by the self-assembled TW molecules. Additionally, glycine, functioning as a cryoprotectant and bulking agent, exhibits high water solubility, further enhancing the colloidal stability of the nanoformulation.^50^

### 3.2 Release Kinetics of Azadirachtin from Composite Biopesticide

Understanding the release kinetics of encapsulated biopesticides is important for establishing a correlation between their liberation rate and efficacy in mortality assays, as well as for predicting the fraction of active ingredients that remain permanently sequestered within the nanocarriers while retaining their pesticidal functionality.^51,52^ Biopesticide formulations exhibiting sustained and gradual release profiles are considered superior to those with rapid release characteristics due to their extended period of bioactivity. This prolonged efficacy is particularly advantageous given that natural pesticide compounds are susceptible to photodegradation, microbial decomposition, and hydrolysis upon environmental exposure.^53–55^ Figure 3 shows that freeze-dried GNP redispersion has a much gradual and slower release of azadirachtin compared to NSE, up to day 7 reaching 68.2 ± 2.1% of the release, while most of NSE diffuses by day 2–3, and up to day 7 reaching 102.8 ± 1.5% calculated from the fitted curve. While 68.2% of the azadirachtin compounds are released, the rest of 31.8% remain in the TW emulsions over 7 days. The release was slow and gradual, sustained for 5+ days to reach a plateau (over 90% of the saturated release level) and has followed an exponential profile, and was close to first-order kinetics. This sustained release profile, the remaining 31.8% encapsulated, is advantageous for killing the insects for longer periods, not only an instant action when the pesticide was directly fed by the insects or delivered systemically inside. This synergistic collaboration of instant release and sustained release can both cover the short and long-term for protecting the crops in the agricultural fields since instantly released azadirachtin can be hydrolyzed, oxidized with pH alteration, digested by microbes with enzymatic mechanisms to lose their functionality within 4 days.^56,57^

**Figure 3:**
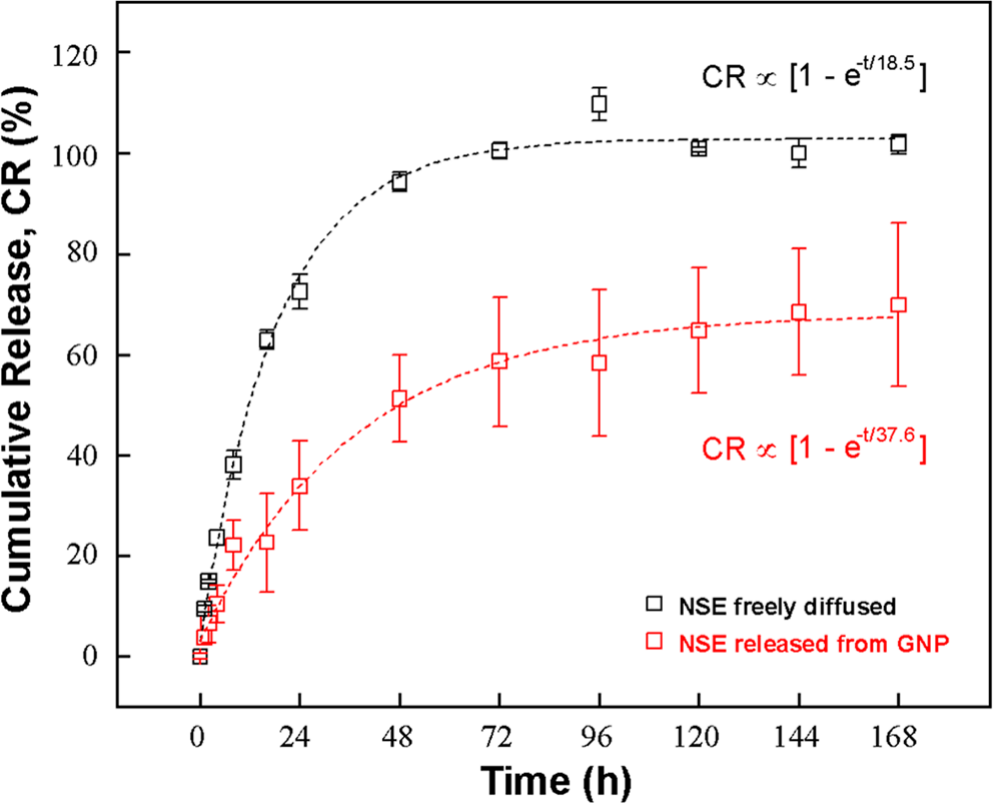
Release kinetics of azadirachtin from GNP in aqueous (water/IPA = 10:1 v/v) solution. The release behavior followed exponential kinetics with cumulative release (CR) ≈ 68.2­[1 – e^–*t*/37.6^] and a coefficient of variance, *r* ^2^ of 0.99. Data represent mean ± standard deviation from triplicate measurements (*n* = 3). Calibration curves are shown in Figure S2.

### 3.3 Stability of Nanobiopesticide Exposed to the UV Irradiation

To further investigate the photostability of the lyophilized GNP composites, we conducted a photodegradation assay. Photostability data under conditions simulating solar irradiation are crucial for determining the shelf life of biopesticide formulations.^58,59^ Plant extracts are known to be susceptible to photolysis and oxidative processes.^60^ Accordingly, we have studied the effect of photodegradation behavior of our novel nanoformulations. Figure 4 shows the comparative photodegradation profiles of encapsulated azadirachtin in the lyophilized GNP formulation versus unformulated NSE powder, as assessed by UV/vis spectroscopy under simulated solar irradiation. Under the experimental conditions, 47.0 ± 3.0% degradation of NSE powder was observed within a four-day period. In contrast, the GNP composite exhibited 41.3 ± 2.0% degradation, while the GNP composite without NSE showed 29.5 ± 3.2% degradation over the same irradiation duration, as evidenced by changes in the corresponding UV/vis spectral peaks.

**Figure 4:**
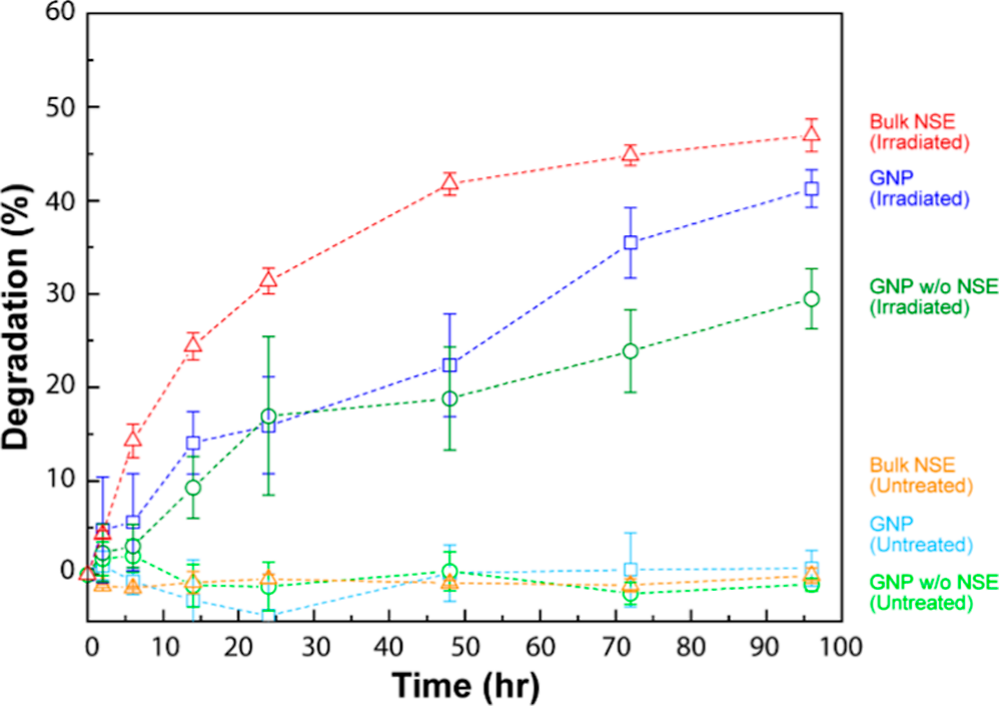
Percent degradation measured from the absorbance intensity loss via UV/vis spectroscopy upon irradiation with UV-AB light in dry conditions. The experiments were performed for GNP composite, a carrier composite (without NSE), NSE powder, and the corresponding controls (dark condition, no irradiation, and room temperature). All experiments were performed at a relative humidity of 50%. Data represent mean ± standard deviation from five replicates (*n* = 5).

The biodegradability of these nanoparticles presents an additional environmental advantage postrelease of the active ingredients into the soil matrix. Comparative analysis reveals that the GNP composite demonstrates a 27.7% enhancement in photostability over a four-day period relative to unformulated NSE powder. We postulate that this reduction in the degradation rate can be attributed to the presence of a sacrificial layer, which induces light scattering effects by encapsulating azadirachtin within a Tween and glycine matrix. The observed photoprotective effect can be explained by Mie and Rayleigh scattering phenomena, whereby a fraction of the incident photonic energy directed at the particle core (where azadirachtin resides) is redistributed to the surrounding encapsulant molecules.^5,61^ This effectively attenuates the photodegradation of the active ingredient.

### 3.4 Survival of *S. frugiperda* against Nanoencapsulated and Bulk Neem Seed Extract

Understanding the efficacy and delivery mechanisms of insecticides is important for developing effective pest control strategies in agriculture.^62^ This can be done in part by evaluating the mortality rates of target pests in response to various insecticide formulations and concentrations. Figure 5a displays the mortality of *S. frugiperda* over 11 days following exposure to six concentrations of GNP, including a water control (0 mg/mL). Figure 5b,c show mortality data for NSE powder (four concentrations) and the GLY + TW control (three concentrations), respectively, over the same 11 day period. The concentrations in Figure 5b,c correspond to the GNP compositions in Figure 5a. Statistical analysis (*p*-values) comparing the efficacy and response time between GNP and the controls is provided in Table S2. LC_50_ values, calculated using a Hill equation fitting model, are summarized in Table S3 and reported by day in Table 1. The LC_50_ data demonstrates consistently higher efficacy and faster response times for GNP compared to NSE. A statistically significant difference (*p* < 0.05) between GNP and bulk NSE was observed up to day 2 (Table S2a), likely due to the increased solubility and mobility of GNP nanoparticles, resulting in a rapid initial killing effect. The release rate appears to decrease after days 2–4. Because of its poor water solubility, NSE interacts with the pests more slowly than GNP.^63^ Furthermore, GNP demonstrated greater efficacy at lower concentrations compared to bulk NSE. For example, on day 7, 10 mg/mL GNP (containing 0.5 mg/mL NSE) resulted in approximately 80 ± 5.8% mortality, whereas 0.5 mg/mL of bulk NSE yielded only 40.6 ± 6.7% to 68.8 ± 12.0% mortality (the third highest NSE concentration, indicated by blue in Figure 5a,b). The enhanced efficacy of GNP may be attributed to its reduced size and increased mobility, facilitating the delivery of azadirachtin into insect organs and tissues. Statistically significant differences (*p* < 0.05) were consistently observed between GLY/TW and NSE (Table S2a). The GLY + TW control exhibited no killing effect except at the highest concentration (950 mg/mL), where mortality reached a maximum of 30%. The lethal effects of these compounds on *S. frugiperda* have not been previously investigated. GLY, an amino acid, is a building block for protein and food, while TW is a common food additive. Previous research reported 0% mortality in *Musca domestica* and *Chrysomya albiceps* exposed to 1% (w/v) TW. The highest concentration of carrier compound used in this study was nearly 100% w/v, 100 times greater than in the previous study, yet even our second highest concentration (10% w/v) did not induce mortality.

**Figure 5:**
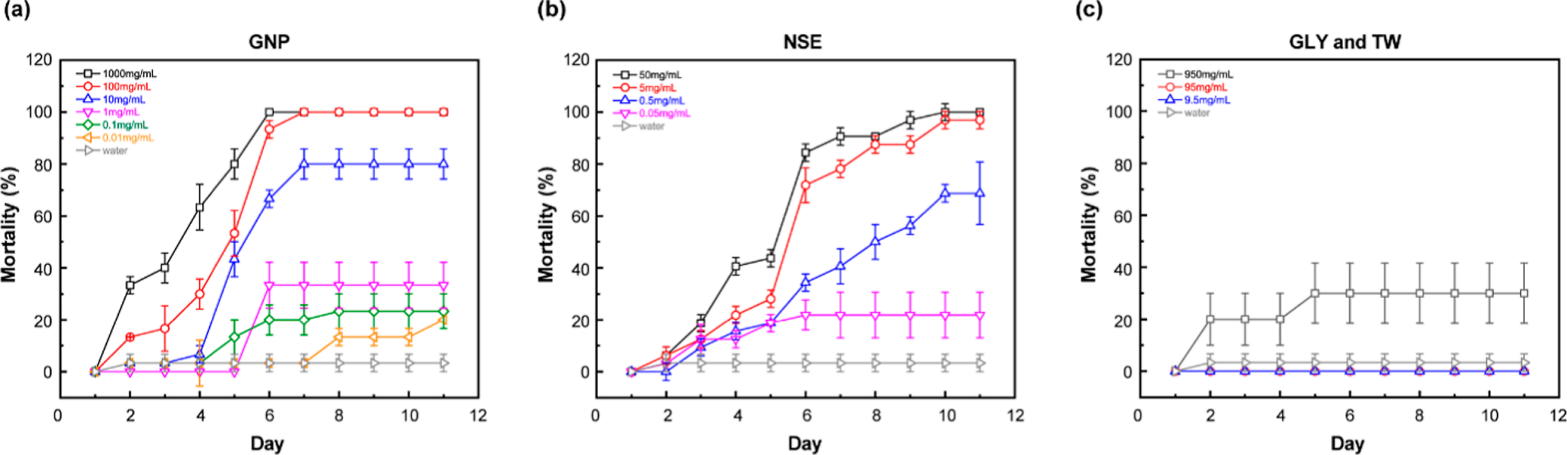
Time-resolved dose–response mortality curves from contact bioassays against *S. frugiperda* larvae exposed to (a) GNP nanoformulation aqueous dispersion, (b) neem seed extract (NSE) aqueous dispersion, and (c) glycine (GLY) + Tween (TW) control aqueous dispersion. Concentration gradients are represented by consistent color schemes across panels. Data represent mean ± standard deviation from three replicates (*n* = 3). Statistical comparisons between groups are provided in Table S2a.

**Table 1:** LC_50_ Values for Contact Mortality Were Estimated Using a Four-Parameter Sigmoidal Dose–Response Model Using the AAT Bioquest LC_50_ Calculator (https://www.aatbio.com/tools/lc50-calculator)^b^

contact mortality		day 4	day 5	day 6	day 7	day 8	day 9	day 10	day 11
NSE encapsulated in GNP	LC_50_ [mg/mL]	7.2	1.4	0.20	0.13	0.15	0.15	0.15	0.16
NSE powder	LC_50_ [mg/mL]	^a^	^a^	1.1	0.86	0.42	0.38	0.24	0.24

a LC_50_ could not be interpolated when average mortality at the highest tested concentration did not exceed 50%.

b “NSE encapsulated in GNP” values were normalized from GNP-based LC_50_ (Table S3) using a 1:19 NSE-to-encapsulant ratio to enable direct comparison with unformulated NSE.

Assessing the impact of insecticides on insect growth and development provides critical insights into their mode of action and overall effectiveness. Figure 6 presents the growth of *S. frugiperda* (measured as length increase) over the same 11 day period as the contact assay in Figure 5, providing further evidence of insecticidal activity and potential antifeedant/growth deterrent effects. These data allow for comparison of growth inhibition across treatments relative to the water control (0 mg/mL). Figure 6a,b demonstrate an inverse relationship between larval length increase and insecticide concentration. Statistical analysis of the length increase trends (Table S2b) reveals no significant difference between GNP and bulk NSE (*p* > 0.05). In contrast, Figure 6c shows that the length data for the GLY and TW controls overlap with the water control, indicating no growth inhibition. A statistically significant difference (*p* < 0.05, except for day 3, *p* = 0.0567 and 0.0530) was observed between the GLY/TW controls and both GNP and bulk NSE, confirming the growth-inhibiting effects of the azadirachtin formulations. Azadirachtin is known to disrupt insect development through mechanisms such as delayed ecdysis and feeding deterrence in pests such as *S. frugiperda*.^64^ The larval stage of *S. frugiperda* (first to sixth instar) typically progresses within 14 days, with pupation potentially initiating afterward. Therefore, monitoring larval length provides a valuable continuous measure of pesticide efficacy prior to pupation. The observed linear growth curve over the 11 day period aligns with previously reported literature.^65^

**Figure 6:**
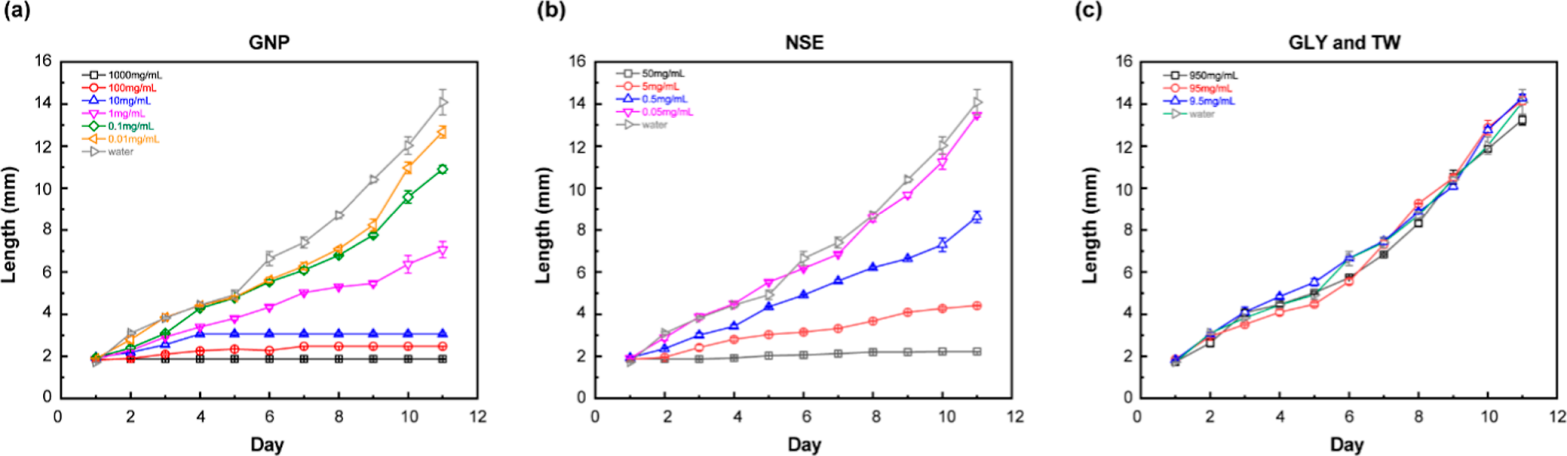
Size analysis from contact assay of *S. frugiperda* (a) GNP dispersion in water, (b) NSE dispersion in water, (c) GLY and TW control dispersion in water. The lines of the same color in (a–c) are comparable to each other, as shown in Figure 5. Data represent mean ± standard deviation from three replicates (*n* = 3). Statistical comparisons between groups are provided in Table S2b.

Comprehensive assessment of insecticide efficacy requires evaluating multiple outcomes, including mortality, growth inhibition, and LC_50_ values, coupled with robust statistical analysis. In this study, mortality, larval length, LC_50_ values, and statistical analysis (*p*-values) collectively demonstrated the enhanced efficacy of nanoencapsulated azadirachtin formulated with GLY and TW. The observed positive correlation between mortality and azadirachtin concentration agrees with previous studies.^66^ Exposure to azadirachtin is known to induce a range of physiological effects, including delayed ecdysis, growth interference, feeding deterrence, sterility, and anatomical aberrations, impacting cells, tissues, nerves, and endocrine systems.^67,68^ To further investigate the effects of GNP under more realistic conditions, a leaf-feeding assay was conducted over the same 11 day period and using the same GNP concentrations as the contact assay (Figure S3). The leaf-feeding assay addressed multiple potential modes of action: mortality due to feeding deterrence/starvation, ingestion and subsequent midgut activity of the nanopesticide, and dermal uptake through contact with the treated leaf surface. Statistical analysis (Table S2c) reveals a significant difference (*p* < 0.05) between the GNP contact and leaf-feeding assays up to day 6 (comparison of Figures 6a and S3), with the same data structure used for both assays across 11 days and at five comparable concentrations. By day 6, the leaf-feeding assay demonstrates greater lethality compared to the contact assay, suggesting enhanced effectiveness as insects consume and digest NSE-coated leaves, leading to higher mortality than surface contact alone. After day 7, however, mortality rates between the two assays converge, with high concentrations achieving near-total lethality across both methods, while lower concentrations display partial efficacy. Corresponding LC_50_ data are presented in Table S4 (plot) and Table S5 (summary values by day). Comparing the LC_50_ values in Table S5 between contact and leaf-feeding assay demonstrated the generally higher toxicity observed in the leaf-feeding assay. This difference is likely attributed to the distinct exposure routes and primary modes of action. The contact assay (2 s exposure) addressed larva exposure via the digestive and respiratory systems, and we used mandible and spiracle sizes as proxies for the size of external openings to those systems; in this assay, entry directly through the integument could not be ruled out but was likely minimal given the short (2 s) exposure and the hydrophobic nature of the insect cuticle, and integument generally. The leaf-feeding assay addressed entry via the digestive system, though entry via spiracles and directly through the integument could not be ruled out. Importantly, the leaf-feeding assay allowed for stronger manifestation of the antifeedant effect of azadirachtin, which is likely to be less apparent in the contact assay where insects are provided an untreated diet.

### 3.5 Morphological and Mechanistic Analysis of Interactions between *S. frugiperda* and Nano Biopesticide

We investigated potential nanoparticle uptake pathways into the internal organs and nervous system of *S. frugiperda* larvae. Specimens were prepared for scanning electron microscopy (SEM) analysis during the contact bioassay. Figure 7 illustrates the morphological overview of the larvae on day 1 (control), day 3 (control), and day 3 (treated with GNP nanoformulation). Detailed examination of the mandibles and spiracles (Figures S4 and S5) confirmed two primary routes of potential nanoparticle ingress. We used the mandibles as a proxy for the size of the oral cavity leading to the postoral digestive system, including the gut, as noted above. The spiracles are the external openings to the respiratory pathway, interface with the tracheal system and lead to all internal tissues.^69,70^ SEM micrographs revealed 10–11 paired spiracles lengthwise on the sides of the larval body.

**Figure 7:**
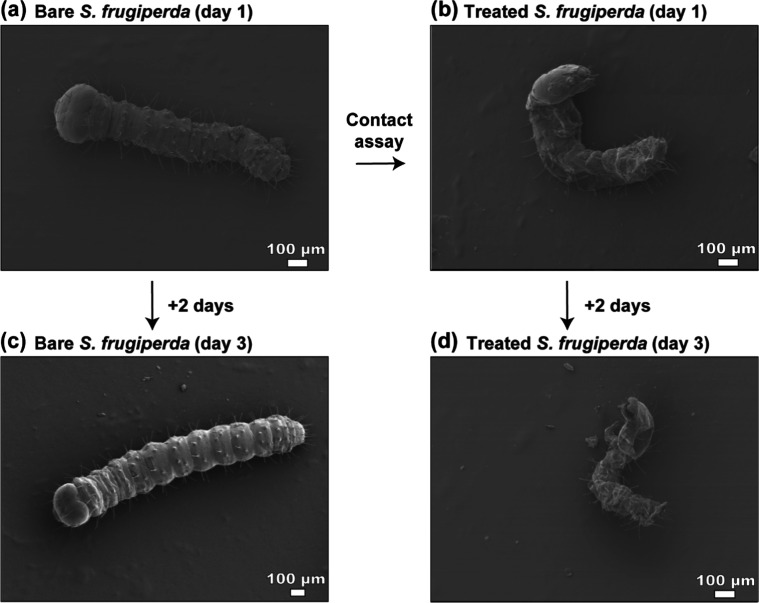
SEM image of *S. frugiperda*, GNP (100 mg/mL) was treated on day 1 using contact assay and larva was grown for 2 days. (a) Bare larva (day 1), (b) treated larva (day 1), (c) bare larva (day 3), (d) treated larva (day 3).

Quantitative analysis of the SEM images yielded dimensional data for the mandibles and spiracles, which are reported in Table 2. Calculations indicated that both mandible and spiracle size exceeded the hydrodynamic diameter of the nanoparticles (reported in Table S1), indicating that nanoformulated insecticide may enter the larva body via either the digestive and respiratory systems. Because mandible size is greater than spiracle size we hypothesize that the volume of nanoparticles entering the larva via the digestive system is greater than the volume entering via the respiratory system.

**Table 2:** Structural and Surface Characteristics of the Mandible and Spiracle of *S. frugiperda* Used in This Study^a^

measurement	mandible	spiracle
long diameter (μm)	82.6 ± 9.0	6.7 ± 1.4
short diameter (μm)	22.2 ± 9.6	4.7 ± 1.0
long × short dimension (μm)	1833.7 ± 86.4	31.5 ± 1.4
function	digestion	respiration

a The average length of filtering protrusions in the spiracle images is 2.3 ± 0.5, with values representing the mean ± standard deviation from three biological replicates (*n* = 3). Mandible length was measured as the distance between the prominent teeth at the larval mouth.

Figure 7 shows pronounced emaciation and cuticular corrugation in the treated larvae at days 1 and 3 postexposure. Correspondingly, Figure S4 reveals nanoparticle deposition and enhanced peritrematic wrinkling in treated specimens. The observed morphological alterations may be attributed to azadirachtin-induced anorexia toward hygroscopic nutriment, resulting in severe desiccation. This dehydration potentially augmented cuticular rugosity and disrupted cellular osmotic homeostasis.^71^

To elucidate the nanoparticle translocation pathways, we incorporated Nile red fluorophore into the initial biopesticidalformulation and conducted contact bioassays as delineated in Figure 8. Supporting Information videos (Figure S6) displaying *z*-stack confocal micrographs provide additional spatial information complementing Figures 8 and S7. Figure 8a–c corroborate our initial hypothesis, demonstrating intense Nile red accumulation along the alimentary tract, originating at the oral cavity. While ubiquitous fluorescence was observed, potentially indicative of spiracular ingress and transcuticular penetration, the mouth and internal digestive system exhibited markedly higher fluorescence intensity. Comparative analysis with a 1:100 dilution (Figure S7) revealed attenuated nanoparticle accumulation, underscoring the concentration-dependent nature of the phenomenon.

**Figure 8:**
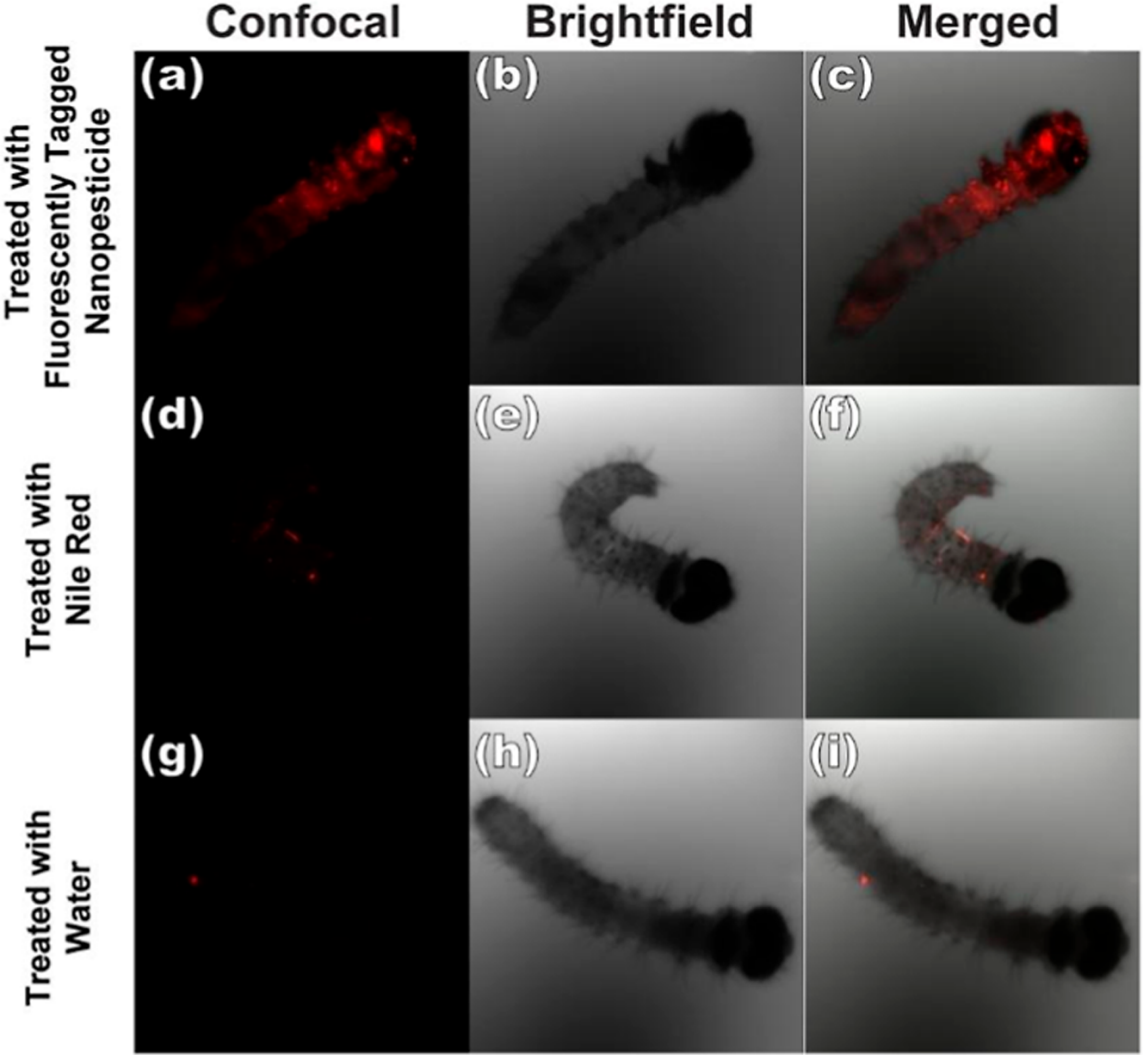
Confocal microscope images of newly hatched *S. frugiperda* (day 1) treated with contact assay. (a–c) Is treated with 100 mg/mL GNP with Nile red (50 μg/mL) dispersion in water, (d–f) is treated with Nile red (50 μg/mL) dispersion in water, and (g–i) is treated with water. Corresponding videos are in Figure S8.

Control experiments utilizing Nile red in aqueous suspension (Figure 8d–f) exhibited significantly reduced fluorophore mobility relative to the nanoparticle-conjugated fluorescence, consistent with the hydrophobic nature of Nile red. Water-only controls (Figure 8g–i) displayed minimal background autofluorescence. Based on these observations, we postulate that the internalized nanoparticles may undergo systemic translocation, potentially accessing a variety of internal organ systems, including endocrine, respiratory, neural, cardiovascular, digestive, excretory, and reproductive tissues.^72–74^ The confocal microscopy data substantiate the capacity of GNP nanoparticles to permeate diverse anatomical compartments within the larval body.

### 3.6 Wetting and Adhesion Characteristics of Composite Biopesticide Solutions on Tomato Leaves

In Figure 9, the static angle of 5 different concentrations of GNP suspension on tomato leaves used for leaf-feeding assay is reported. The coating behavior corresponds to the hydrophilicity and the static angle profiles between the substrate and the solutes in water.^75–77^ The 0.1 mg/mL GNP concentration was used as a standardized formulation for nanoparticle preparation and release profiling studies, and may represent a practical concentration range for field application. For the wetting analysis (Figure 9), GNP solutions were tested from 0.01 to 100 mg/mL to simulate field-relevant droplet evaporation behavior. The static angle of water droplet on the tomato leaf surface was measured as 99.0 ± 1.6°. Both 0.01 and 0.1 mg/mL concentrations initially exhibited contact angles similar to water (∼99°), reflecting minimal wetting at low solute levels. As concentration increased, contact angle progressively decreased, illustrating how drying enhances surface coverage via glycine-Tween mediated coating. This behavior aligns with the concentration-dependent trends observed in both contact and feeding bioassay. It is known that leaf surfaces are naturally hydrophobic owing to the natural wax layers and microroughness.^40,78^ The static angle from the lowest concentration from Figure 9 overlaps in the range of the water droplets with standard error, but it greatly starts to drop from 1 mg/mL scale. In the simulated field, as the solvent dries over time, the solutes can either be coated or moved away from the surface depending on the hydrophilicity. As the static angle from the highest concentration has been near 60°, most of the nanoparticles are expected to be coated on the leaf surface. The biopesticides can either be directly sprayed on the insects or cover the plant surfaces to protect against the pests. The contact assay and the leaf-feeding assay are correlated with those cases, respectively. GLY is highly water-soluble and hydrophilic, and TW is amphiphilic and strongly bound to NSE compounds and can stick to the leaf surface simultaneously. GLY and TW both have contributed to increasing the wettability to make GNP nanoparticles to adhere on the tomato leaf substrate.^79^

**Figure 9:**
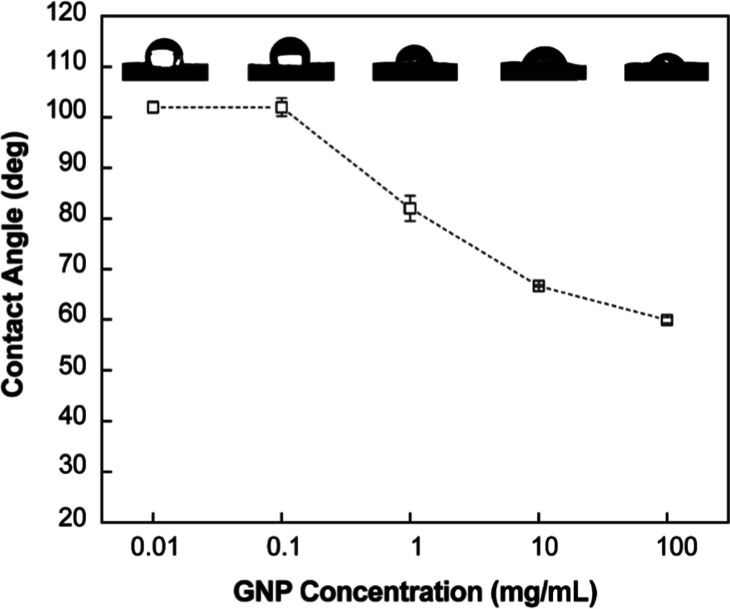
Static contact angle of GNP aqueous solutions on tomato leaves at varying concentrations. The observed decrease from 99.0 ± 1.6° to approximately 60° indicates enhanced wettability, as glycine and Tween 80 progressively replace water during droplet drying, promoting foliar coating and adhesion relevant to pesticidal applications. Measurements were performed in triplicate (*n* = 3).

The improved wetting and adhesion at higher GNP concentrations can be attributed to the synergistic effects of the hydrophilic glycine functionalization and the amphiphilic Tween 80 surfactant used in the formulation. These components help bridge the hydrophobic–hydrophilic interface between the leaf surface and aqueous suspension. From an application standpoint, the enhanced wetting and adhesion at higher concentrations indicates that more concentrated GNP formulations would likely achieve better leaf surface coverage and retention when sprayed in field conditions, potentially improving the efficacy and persistence of the nanopesticide treatment.^40^ These findings have important implications for the interaction of nanopesticides with plant waxes and foliar adhesion: (i) the ability of higher GNP concentrations to overcome leaf hydrophobicity suggests that the nanopesticide formulation can effectively penetrate and interact with the epicuticular wax layer, which is crucial for pesticide retention and efficacy. (ii) The concentration-dependent wetting behavior indicates that optimizing the GNP concentration is critical for achieving the desired balance between spread and adhesion on the leaf surface. This could allow for more precise control over pesticide distribution and persistence. (iii) The role of the amphiphilic Tween 80 in enhancing wettability highlights the importance of surfactant selection in nanopesticide formulations for overcoming the barrier posed by leaf waxes and promoting strong foliar adhesion.

## 4 Conclusions

This study demonstrates the successful development and comprehensive characterization of a novel glycine-based nanoformulation biopesticide encapsulating azadirachtin derived from neem seed extract. The nanoformulation exhibited superior physicochemical properties and enhanced biological efficacy against *S. frugiperda* larvae compared to bulk azadirachtin, representing a significant advancement in biopesticide technology.

In vitro release kinetics studies elucidated the controlled release behavior of the nanoformulation. The release profile followed an exponential model (*r* ^2^ = 0.99) with a time constant of 37.6 h, achieving a cumulative release of 68.2 ± 2.1% over a 7 day period. Notably, 31.8% of the active ingredient remained encapsulated, potentially contributing to prolonged efficacy. This sustained release characteristic aligns with the observed mortality kinetics in bioassays, where efficacy plateaued after approximately 5 days.

Photostability assays under simulated solar irradiation demonstrated the nanoformulation’s enhanced resistance to UV-induced degradation. The nanoformulation exhibited a 27.7% reduction in degradation rate compared to unformulated neem seed extract over a 4 day period. This improved photostability is attributed to the light scattering effects of the nanostructure and the presence of a sacrificial composite carrier layer, potentially extending the environmental persistence of the biopesticide.

Bioefficacy evaluations through LC_50_ analysis revealed markedly superior pesticidal activity of the nanoformulation. At day 7 post-treatment, the LC_50_ for nanoencapsulated azadirachtin was determined to be 0.13 mg/mL, compared to 0.86 mg/mL for bulk azadirachtin, representing a 6.6-fold increase in potency. This enhanced efficacy is likely attributable to improved bioavailability and cellular uptake facilitated by the nanocarrier system.

Mechanistic investigations into uptake pathways utilized confocal microscopy with Nile red-tagged nanoparticles. These studies revealed systemic translocation of the nanoformulation throughout larval tissues, with primary ingress occurring via the oral cavity and alimentary canal. Scanning electron microscopy further elucidated potential uptake routes, identifying the oral cavity and spiracles as key entry points for the nanoparticles.

Foliar adhesion properties were assessed through static contact angle measurements on tomato leaf surfaces. The nanoformulation demonstrated concentration-dependent improvements in wettability, with contact angles decreasing from 99.0° ± 1.6° for water to approximately 60° at 100 mg/mL nanoformulation concentration. This enhanced wetting behavior is attributed to the synergistic effects of hydrophilic glycine functionalization and the amphiphilic nature of Tween 80 in the formulation.

In essence, this glycine-based nanoencapsulation strategy presents a promising approach for the development of next-generation biopesticides with enhanced stability, bioavailability, and efficacy. The controlled release properties, improved photostability, and superior wetting characteristics of the formulation offer significant advantages for sustainable pest management in organic agriculture. While the present study focused on laboratory-scale validation, future work may involve evaluating batch reproducibility at larger scales, exploring cost-effective alternatives to the nanoprecipitation process, and assessing long-term storage stability. Field trials, particularly in horticultural and organic farming systems, will be essential to confirm the formulation’s efficacy under realistic environmental conditions. Consideration of factors such as environmental variability, application methods, and formulation robustness during field deployment will further support the translation of this platform into practical agricultural use.

## Supplementary Material





## Notes

Schematic protocols used for insect mortality analysis, additional information about particle preparation (DLS), calibration plot for release kinetics test, mortality plot from leaf-feeding assay, additional SEM micrographs for *S. frugiperda*, the video clip of confocal microscope stack-wise by *z*-axis, additional confocal microscope image, statistical data obtained from mortality results, interpolation of contact and leaf-feeding assay mortality results for LC_50_ (PDF)
Video (ZIP)

## Abbreviations

IPM: integrated pest management
TEM: transmission electron microscopy
LC_50_: median lethal concentration
DI: deionized
GLY: glycine
TW: Tween 80
NSE: neem seed extract
IPA: isopropanol
DLS: dynamic light scattering
GNP: glycine nanopesticide
SEM: scanning electron microscopy
PDI: polydispersity index
CR: cumulative release
