# Supplementary material for: Tailoring Biopesticides: Amphiphile-Assisted Nanoprecipitation of Azadirachtin within a Glycine Matrix for Sustainable Agriculture, Enhanced Stability, and Larvicidal Efficacy against Fall Armyworm
Source: ACS Omega. 2025 Aug 19;10(34):38766–79. doi: 10.1021/acsomega.5c04028 (PMC12409566; doi:10.1021/acsomega.5c04028)
Supplement: Supplementary file 1 [file ao5c04028_si_001.pdf]

Tailoring Biopesticides: Amphiphile-Assisted Nanoprecipitation of Azadirachtin within a Glycine Matrix for Sustainable Agriculture, Enhanced Stability, and Larvicidal Efficacy against Fall Armyworm

*Michael Bae [a, *], Amanda Lewis [b, *], Shuhao Liu [c], Yashwanth Arcot [a], Yu-Ting Lin [a], Laxmi S Viswanadha [d], Julio S Bernal [e], Mustafa Akbulut [a, f, **], and Luis Cisneros-Zevallos [b, **]*

[a] Artie McFerrin Department of Chemical Engineering, Texas A&M University, College Station, TX 77843, USA
[b] Department of Horticultural Science, Texas A&M University, College Station, TX 77843, USA
[c] Jasper Department of Chemical Engineering, The University of Texas at Tyler, Tyler, TX 75799, USA
[d] J. Mike Walker '66 Department of Mechanical Engineering, Texas A&M University, College Station, TX 77843, USA
[e] Department of Entomology, Texas A&M University, College Station, TX 77843, USA
USA
[f] Department of Materials Science and Engineering, Texas A&M University, College Station, TX 77843, USA

[*] Both authors contributed equally to this work.
[**] Correspondence and requests for materials should be addressed to MA and LCZ. (Email: makbulut@tamu.edu, lcisnero@tamu.edu).

TABLE OF CONTENTS

Scheme S1. Contact Assay.

Scheme S2. Feeding Assay.

Figure S1. The intensity-averaged particle size (hydrodynamic diameter) distribution of (a) as-prepared, (b) after freeze-drying (c)-(h) preliminarily changing the weight ratio between TW and NSE.

Figure S2. Calibration plot for release kinetics test.

Figure S3. Leaf-feeding assay with GNP with *S. frugiperda* for the application in the field.

Figure S4. SEM image of *S. frugiperda* spiracle morphology.

Figure S5. SEM image of control *S. frugiperda* (day 1), (a) upside-down posture (b) head part (c) mandible part.

Figure S6. Confocal microscope video of newly hatched *S. frugiperda* (day 1) treated with contact assay. (a) is treated with 100 mg/ml GNP with Nile red (50 μg/ml) dispersion in water, (b) is treated with 1mg/ml GNP with Nile red (0.5 μg/ml) dispersion in water (1/100 dilution), (c) is treated with Nile red (50 μg/ml) dispersion in water, and (d) is treated with water. Double-clicking will allow to play the file.

Figure S7. Confocal microscope image of newly hatched *S. frugiperda* (day 1) treated with contact assay. (a)-(c) is treated with 1mg/mL GNP with Nile red (0.5 μg/mL) dispersion in water (100:1 dilution).

Table S1. Particle characterization data of different mixing ratios for the emulsion, between TW and NSE.

Table S2. Difference report (p-value) for (a) contact assay mortality (b) size analysis from contact assay (c) between two assays.

Table S3. Table S3. Interpolated LC_{50} of GNP for contact assay using the AAT Bioquest LC_{50} Calculator. GNP did not exceed 50% mortality before Day 4, and NSE did not exceed 50% mortality before Day 6. Each data point represents the average of three replicates. The calculation program is available at https://www.aatbio.com/tools/lc50-calculator.

Table S4. Table S4. Interpolated LC_{50} values for the leaf-feeding assay using the AAT Bioquest LC_{50} Calculator. GNP did not exceed 50% mortality before Day 3. Each data point represents the average of three replicates. The calculation program is available at https://www.aatbio.com/tools/lc50-calculator."

Table S5. LC_{50} values for leaf-feeding mortality. 'NSE encapsulated in GNP' was quantified by normalizing the GNP LC_{50} value (Table S4) using an NSE-to-encapsulant ratio of 1:19, allowing for a lethality comparison between the contact and leaf-feeding assays.

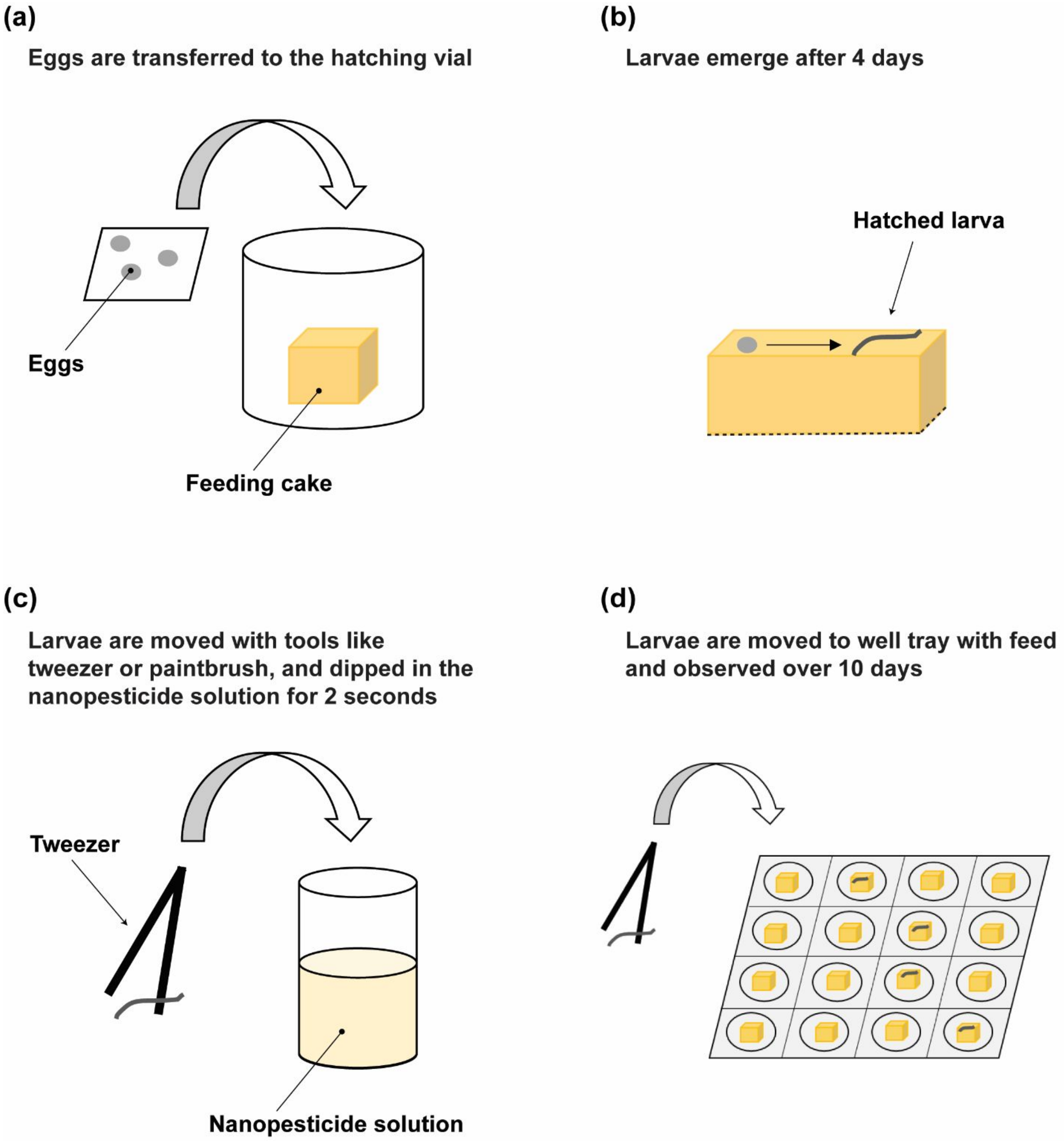

Scheme S1. Contact assay.

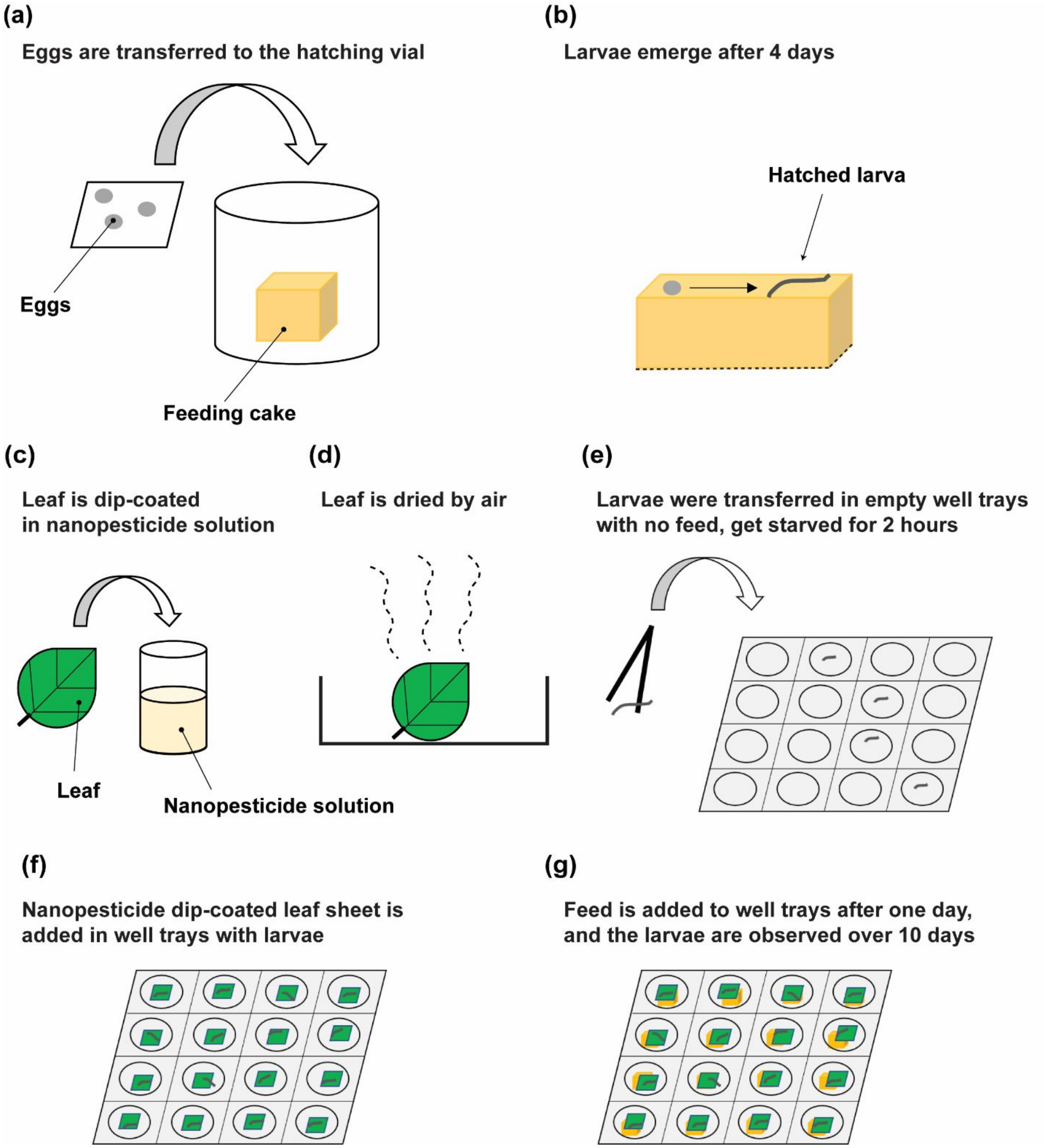

Scheme S2. Leaf-feeding assay.

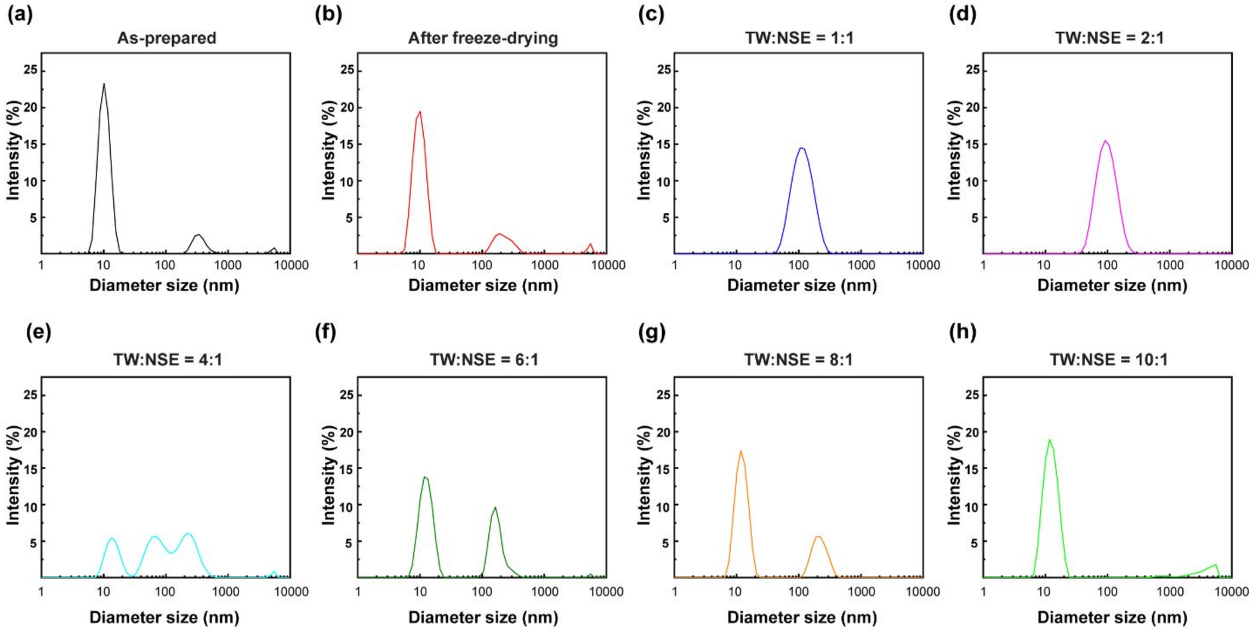

Figure S1. The intensity-averaged particle size (hydrodynamic diameter) distribution of (a) as-prepared, (b) after freeze-drying (c)-(h) preliminarily changing the weight ratio between TW and NSE.

(a)

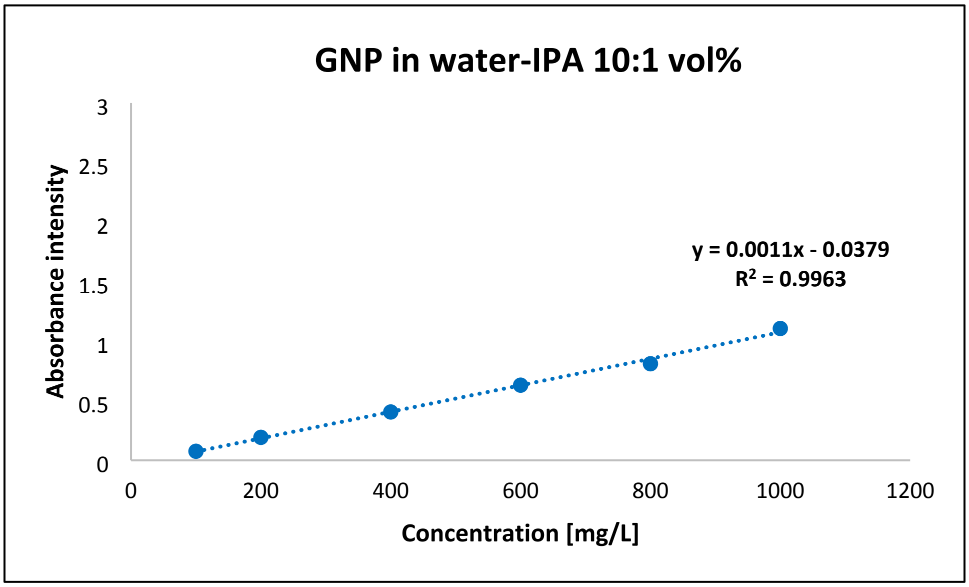
GNP in water-IPA 10:1 vol%
Absorbance intensity
Concentration [mg/L]
y = 0.0011x - 0.0379
R² = 0.9963
0
0.5
1
1.5
2
2.5
3
0
200
400
600
800
1000
1200

(b)

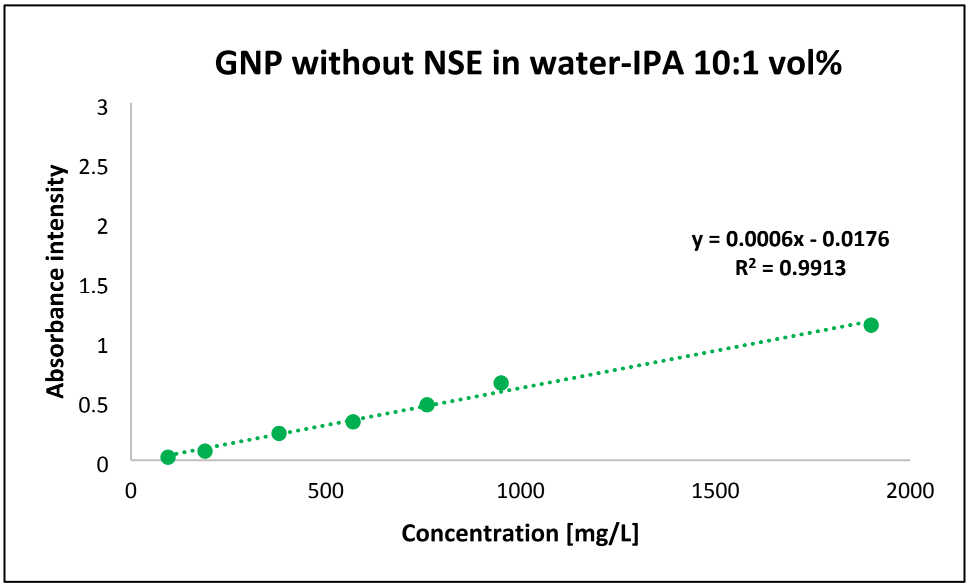
GNP without NSE in water-IPA 10:1 vol%
Absorbance intensity
Concentration [mg/L]
y = 0.0006x - 0.0176
R² = 0.9913
0
0.5
1
1.5
2
2.5
3
0
500
1000
1500
2000

(c)

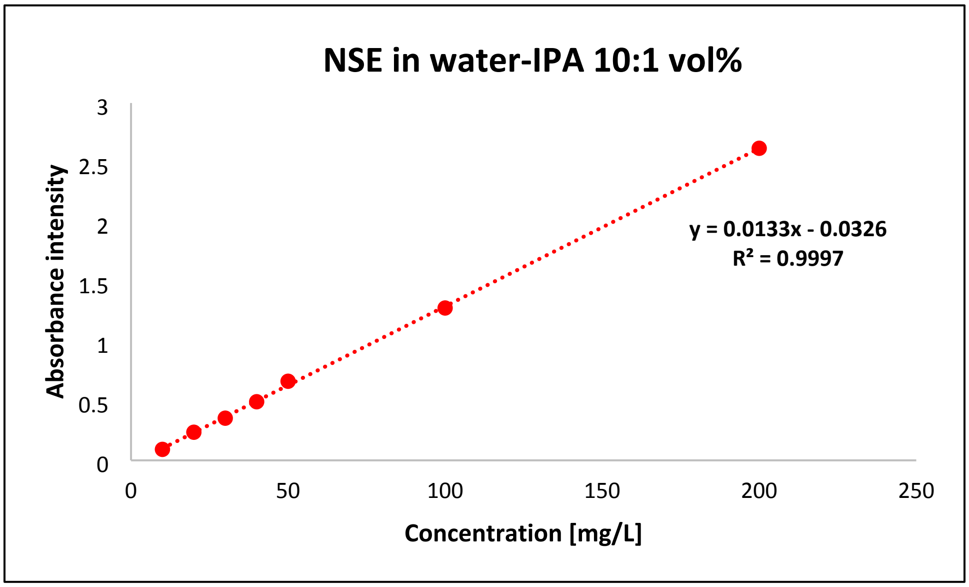

Figure S2. Calibration plots for absorbance-based release kinetics analysis in water-IPA (10:1 vol%) solvent system. (a) Calibration curve of GNP with NSE encapsulated, (b) calibration curve of GNP without NSE, and (c) calibration curve of NSE alone. Linear regression fits and R^2 values are included in each plot for quantitative reference.

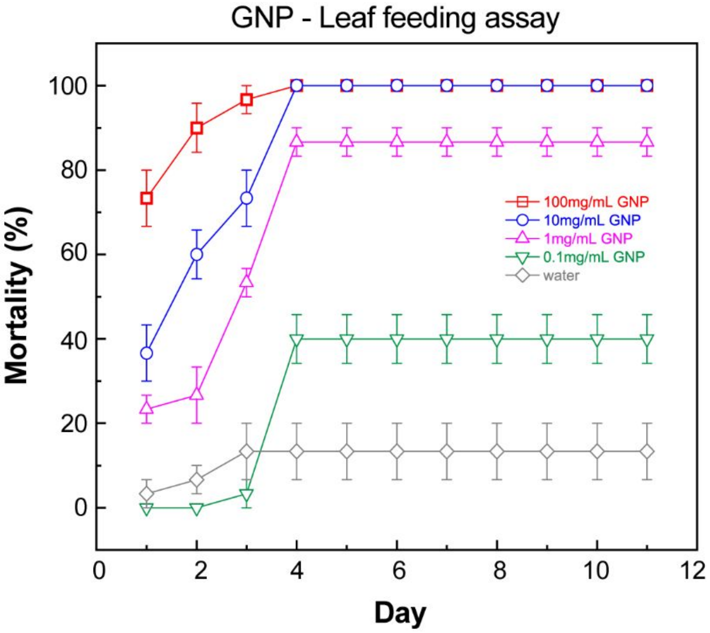

Figure S3. Leaf-feeding assay with GNP using *S. frugiperda* to simulate field application conditions (n = 3). Statistical comparisons between contact and leaf-feeding assay are provided in Table S2-c.

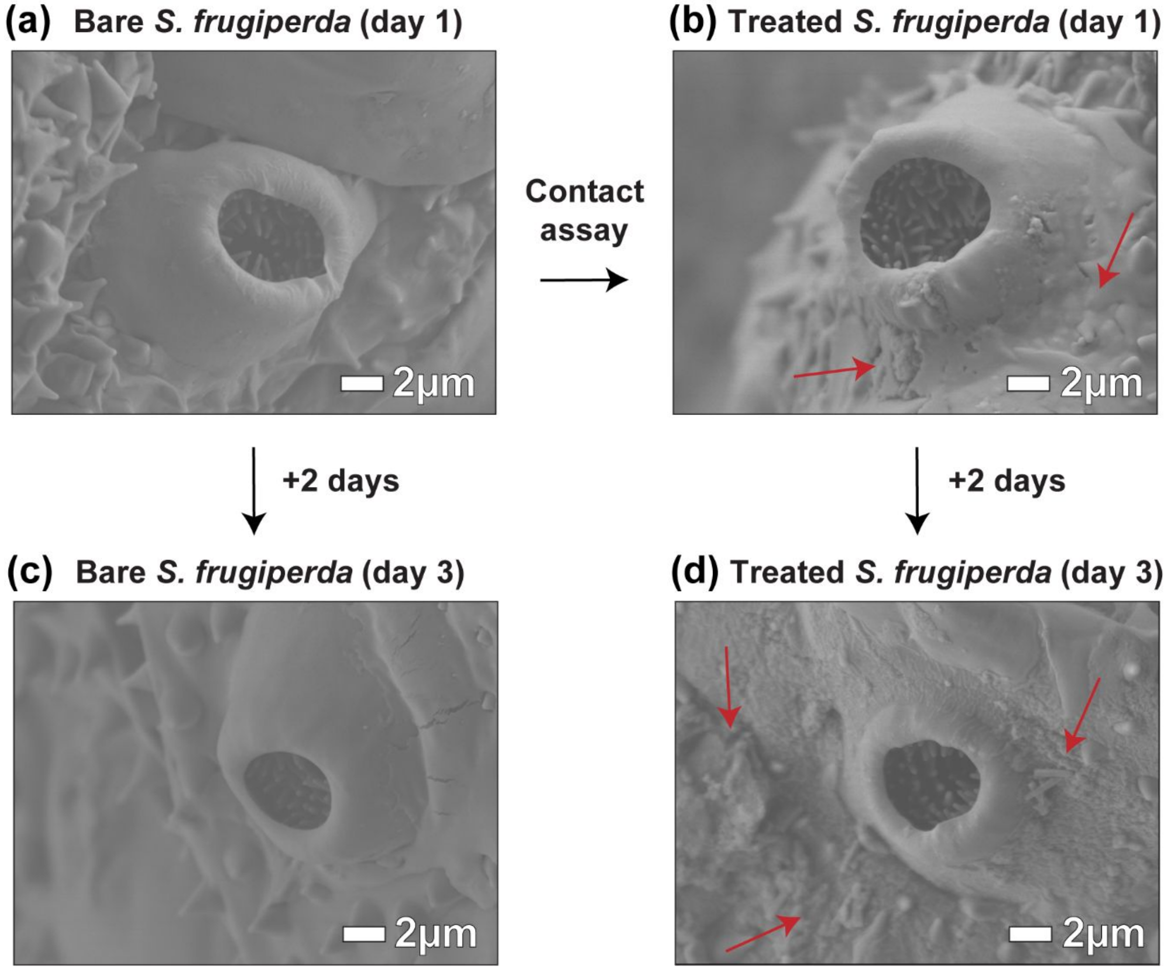

Figure S4. SEM image of *S. frugiperda* spiracle morphology, GNP (100 mg/ml) was treated on day 1 using contact assay and larva was grown for 2 days. (a) control larva (day 1), (b) treated larva (day 1), (c) control larva (day 3), (d) treated larva (day 3).

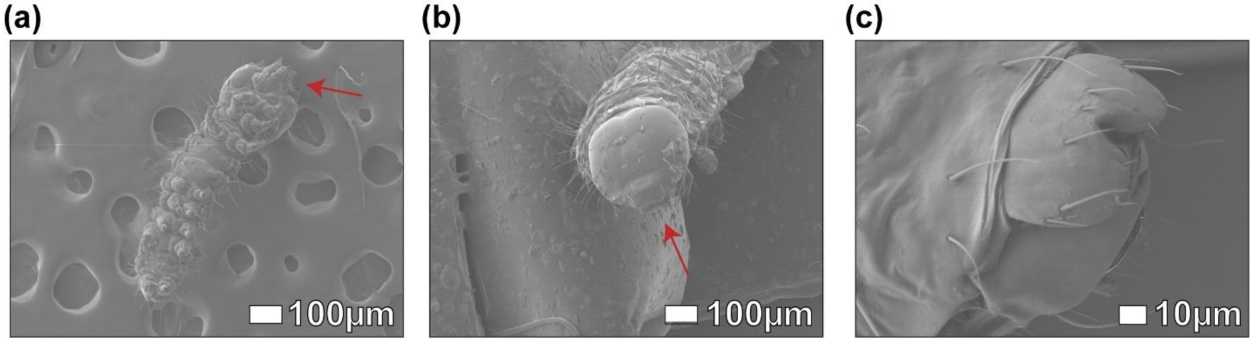

Figure S5. SEM image of control *S. frugiperda* (day 1), (a) upside-down posture (b) head part (c) mandible part.

(a)<br>Fig S6-a.mp4	(b)<br>Fig S6-b.mp4
(c)<br>Fig S6-c.mp4	(d)<br>Fig S6-d.mp4

Figure S6. Confocal microscope video of newly hatched *S. frugiperda* (day 1) treated with contact assay. (a) is treated with 100 mg/ml GNP with Nile red (50 μg/ml) dispersion in water, (b) is treated with 1mg/ml GNP with Nile red (0.5 μg/ml) dispersion in water (1/100 dilution), (c) is treated with Nile red (50 μg/ml) dispersion in water, and (d) is treated with water. Double-clicking will allow to play the file.

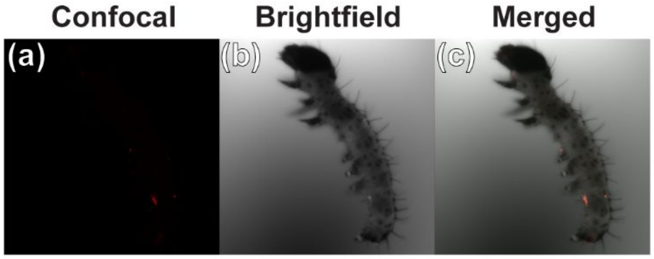

Figure S7. Confocal microscope image of newly hatched *S. frugiperda* (day 1) treated with contact assay. (a)-(c) is treated with 1 mg/mL GNP with Nile red (0.5 μg/mL) dispersion in water (100:1 dilution).

Table S1. Particle characterization data of different mixing ratios between Tween 80 and neem seed extract (NSE). All values represent mean ± standard deviation from three independent measurements (n = 3).

Mixing ratio (TW:NSE)	Organic phase (Isopropanol 10ml)		Water phase (Water 100ml)	Z-Average (nm)	PDI	Azadirachtin wt% after freeze-drying
	Tween 80 (TW)	Neem seed extract (NSE)*	Glycine (GLY)			
1:1	0.05g	0.05g	-	105.7 ± 3.7	0.18 ± 0.01	10.5
2:1	0.10g	0.05g	-	85.6 ± 2.8	0.26 ± 0.02	7.0
4:1	0.20g	0.05g	-	115.3 ± 43.3	0.39 ± 0.03	4.2
6:1	0.30g	0.05g	-	140.0 ± 70.4	0.36 ± 0.10	3.0
8:1	0.40g	0.05g	-	72.6 ± 15.3	0.27 ± 0.03	2.3
10:1	0.50g	0.05g	-	13.6 ± 0.7	0.25 ± 0.04	1.9
10:1 (As-prepared)	0.50g	0.05g	0.45g	47.6 ± 24.8	0.22 ± 0.04	1.1
10:1 (After freeze-drying)	0.50g	0.05g	0.45g	39.6 ± 29.1	0.22 ± 0.02	1.1

* NSE has pure azadirachtin 21wt% and the rest are other limonoid derivatives, polyphenolics, lignins, fatty acid esters, and lipids.

Table S2. Difference report (*p*-value) for (a) contact assay mortality (b) size analysis from contact assay (c) between two assays.

(a)

GNP vs NSE	Day 1	Day 2	Day 3	Day 4	Day 5	Day 6	Day 7	Day 8	Day 9	Day 10	Day 11
p value	-*	0.0019	0.9553	0.7293	0.2102	0.5954	0.8188	0.7732	0.6967	0.6967	0.5047
GNP vs GLY+TW	Day 1	Day 2	Day 3	Day 4	Day 5	Day 6	Day 7	Day 8	Day 9	Day 10	Day 11
p value	-*	0.0079	0.0048	0.0032	0.0007	0.0007	0.0028	0.0020	0.0020	0.0020	0.0017
NSE vs GLY+TW	Day 1	Day 2	Day 3	Day 4	Day 5	Day 6	Day 7	Day 8	Day 9	Day 10	Day 11
p value	-*	0.0444	0.0048	0.0003	0.00001	0.0003	0.0007	0.0008	0.0020	0.0020	0.0044

* Day 1 are all 0 mortality in the beginning

(b)

GNP vs NSE	Day 1	Day 2	Day 3	Day 4	Day 5	Day 6	Day 7	Day 8	Day 9	Day 10	Day 11
p value	0.7710	0.5084	0.3901	0.6474	0.1310	0.2149	0.2219	0.1027	0.1212	0.3385	0.2829
GNP vs GLY+TW	Day 1	Day 2	Day 3	Day 4	Day 5	Day 6	Day 7	Day 8	Day 9	Day 10	Day 11
p value	0.0194	0.0020	0.0567	0.0063	0.0085	0.0054	0.0039	0.0053	0.0372	0.0176	0.0222
NSE vs GLY+TW	Day 1	Day 2	Day 3	Day 4	Day 5	Day 6	Day 7	Day 8	Day 9	Day 10	Day 11
p value	0.0485	0.0035	0.0530	0.0070	0.0085	0.0027	0.0017	0.0028	0.0159	0.0041	0.0061

(c)

GNP contact vs feeding	Day 1	Day 2	Day 3	Day 4	Day 5	Day 6	Day 7	Day 8	Day 9	Day 10	Day 11
p value	-*	0.0004	0.0002	0.0010	0.0010	0.0216	0.0674	0.0750	0.0750	0.0750	0.0750

* Day 1 are all 0 mortality in the beginning for both assays

Table S3. Interpolated LC_{50} of GNP for contact assay using the AAT Bioquest LC_{50} Calculator. GNP did not exceed 50% mortality before Day 4, and NSE did not exceed 50% mortality before Day 6. Each data point represents the average of three replicates. The calculation program is available at https://www.aatbio.com/tools/lc50-calculator.

**Contact Assay**	**Day**	**Plot**	**Equation fitting with** $Y = Min + \frac{Max - Min}{1 + (\frac{X}{LC_{50}})^{Hill\ coefficient}}$
GNP	4	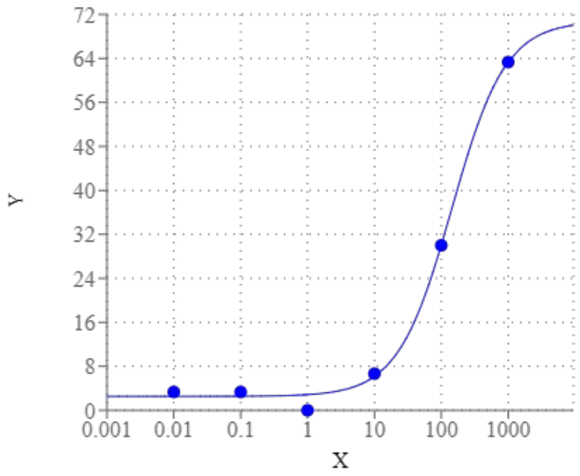	$Y = 2.5321 + \frac{70.7535 - 2.5321}{1 + (\frac{X}{143.4866})^{-1.0825}}$

	5	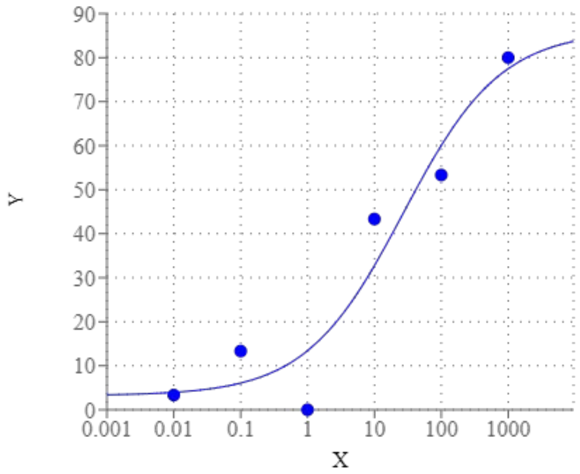	$Y = 3.212 + \frac{86.1316 - 3.212}{1 + (\frac{X}{27.0172})^{-0.5957}}$
	6	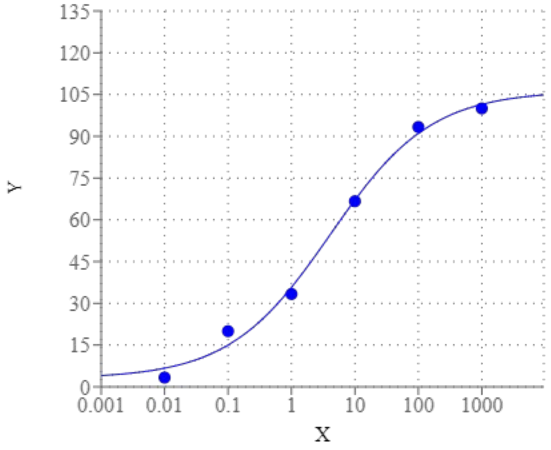	$Y = 2.9775 + \frac{106.301 - 2.9775}{1 + (\frac{X}{4.067})^{-0.5472}}$
	7	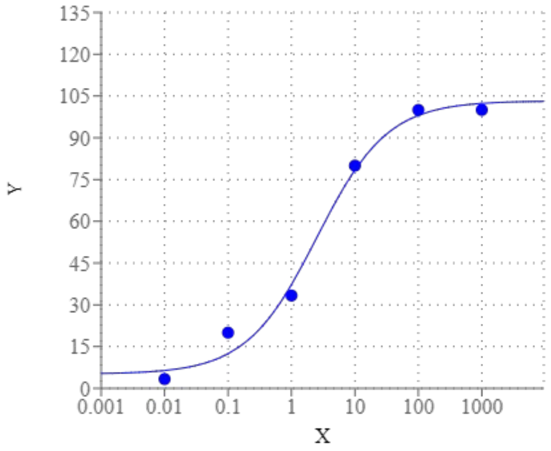	$Y = 5.1332 + \frac{103.2359 - 5.1332}{1 + (\frac{X}{2.5099})^{-0.7818}}$

	8	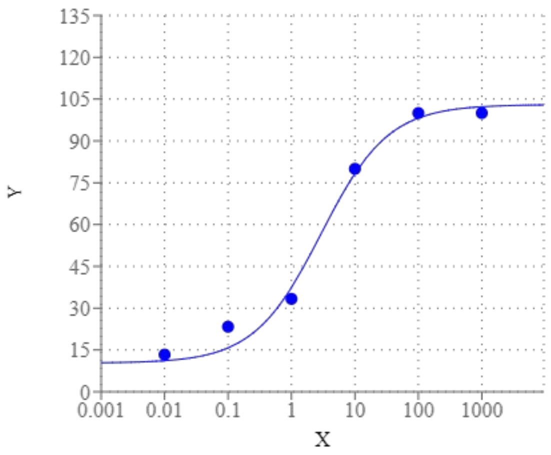	$Y = 10.2909 + \frac{103.0212 - 10.2909}{1 + (\frac{X}{2.9252})^{-0.8219}}$
	9	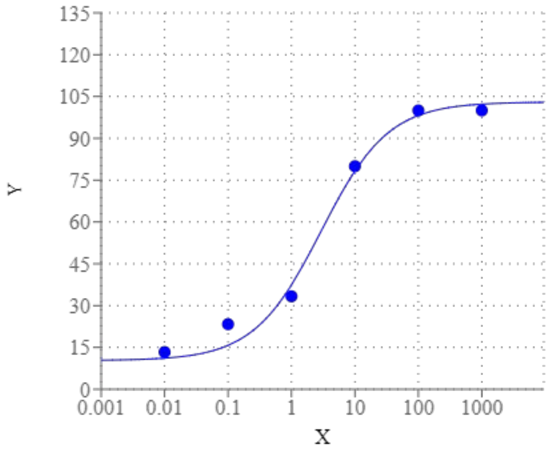	$Y = 10.2909 + \frac{103.0212 - 10.2909}{1 + (\frac{X}{2.9252})^{-0.8219}}$
	10	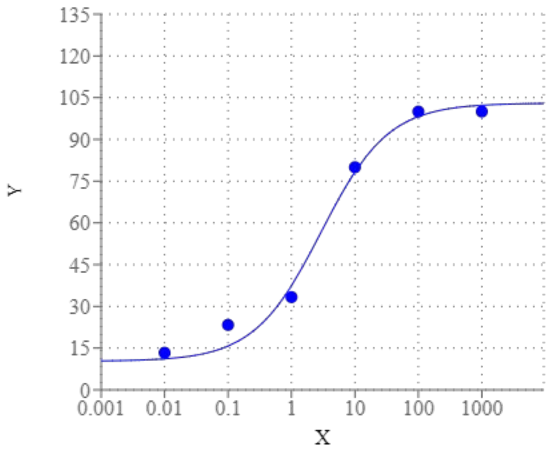	$Y = 10.2909 + \frac{103.0212 - 10.2909}{1 + (\frac{X}{2.9252})^{-0.8219}}$

	11	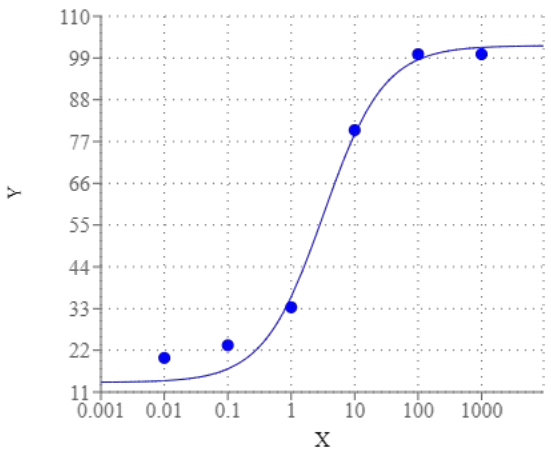	$Y = 13.5524 + \frac{102.244 - 13.5524}{1 + (\frac{X}{3.241})^{-0.9141}}$
NSE Powder	6	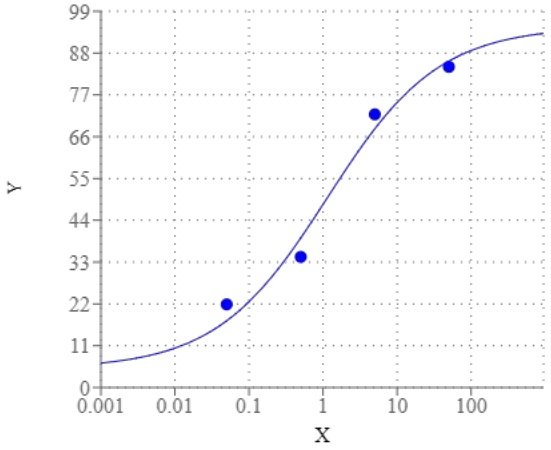	$Y = 4.9415 + \frac{94.9596 - 4.9415}{1 + (\frac{X}{1.1443})^{-0.5793}}$
	7	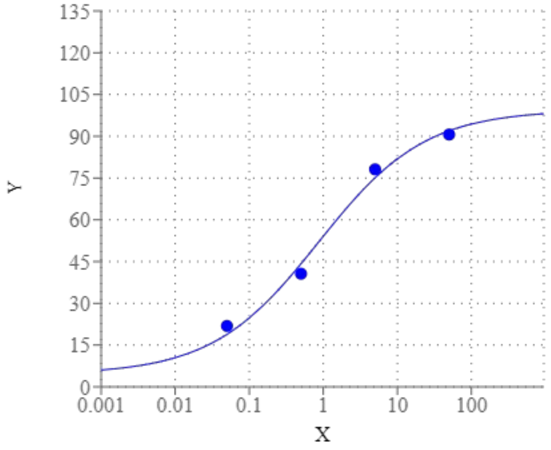	$Y = 4.4866 + \frac{99.4285 - 4.4866}{1 + (\frac{X}{0.8617})^{-0.6055}}$

	8	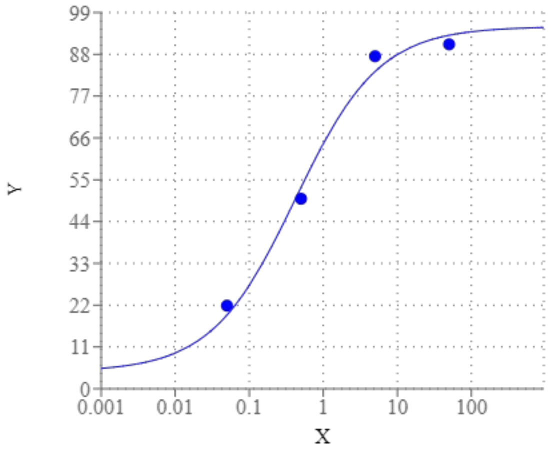	$Y = 4.4873 + \frac{95.2877 - 4.4873}{1 + (\frac{X}{0.4187})^{-0.7658}}$
	9	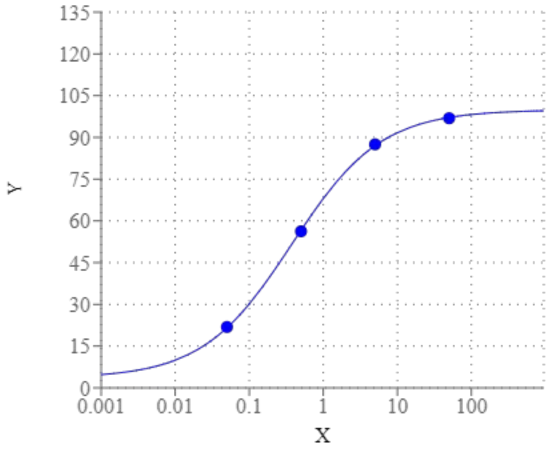	$Y = 3.4431 + \frac{99.8747 - 3.4431}{1 + (\frac{X}{0.3762})^{-0.7236}}$
	10	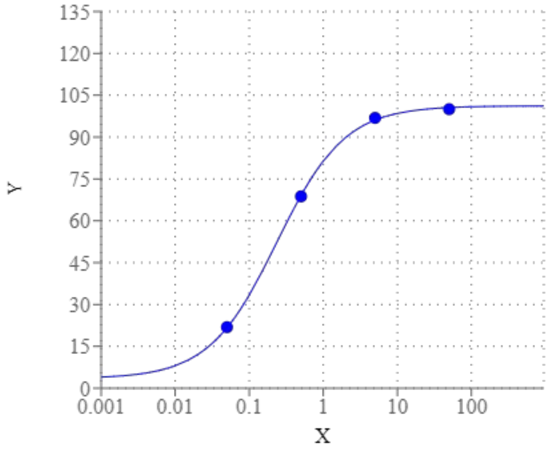	$Y = 3.4408 + \frac{101.1823 - 3.4408}{1 + (\frac{X}{0.2358})^{-0.9499}}$

	11	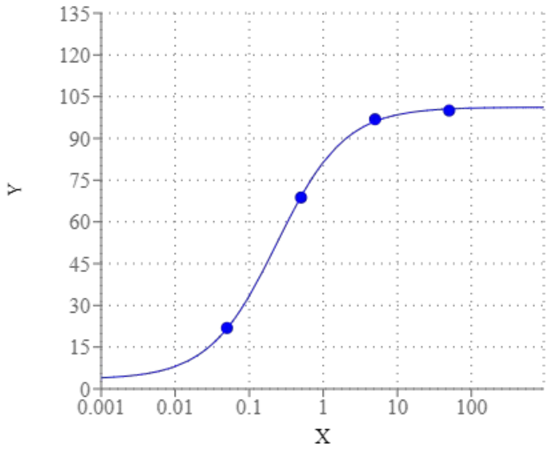	$Y = 3.4408 + \frac{101.1823 - 3.4408}{1 + (\frac{X}{0.2358})^{-0.9499}}$

Table S4. Interpolated LC_{50} values for the leaf-feeding assay using the AAT Bioquest LC_{50} Calculator. GNP did not exceed 50% mortality before Day 3. Each data point represents the average of three replicates. The calculation program is available at https://www.aatbio.com/tools/lc50-calculator."

**Leaf-Feeding Assay**	**Day**	**Plot**	**Equation fitting with** $Y = Min + \frac{Max - Min}{1 + (\frac{X}{LC_{50}})^{Hill\ coefficient}}$
GNP	2	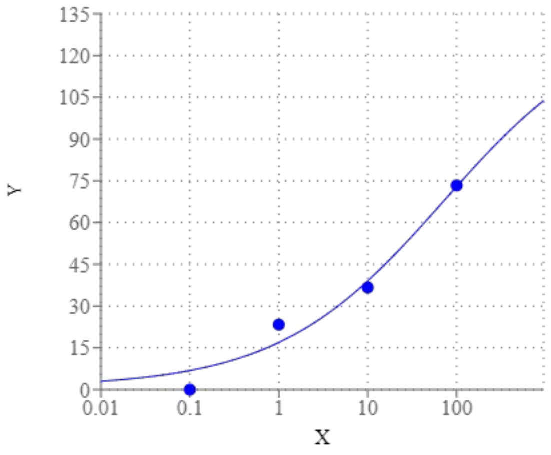	$Y = 0.9233 + \frac{134.4304 - 0.9233}{1 + (\frac{X}{71.2819})^{-0.4673}}$

	3	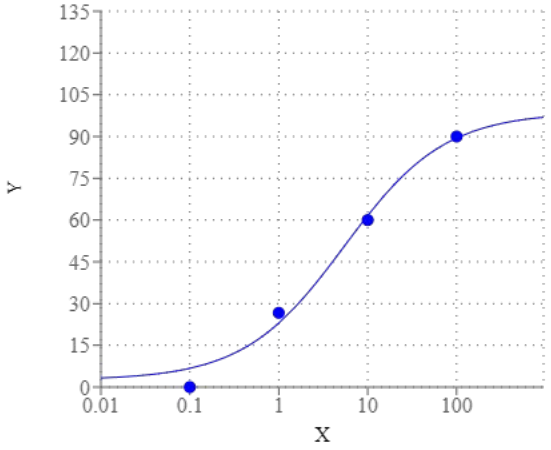	$Y = 2.5377 + \frac{98.6943 - 2.5377}{1 + (\frac{X}{5.4618})^{-0.7705}}$
	4	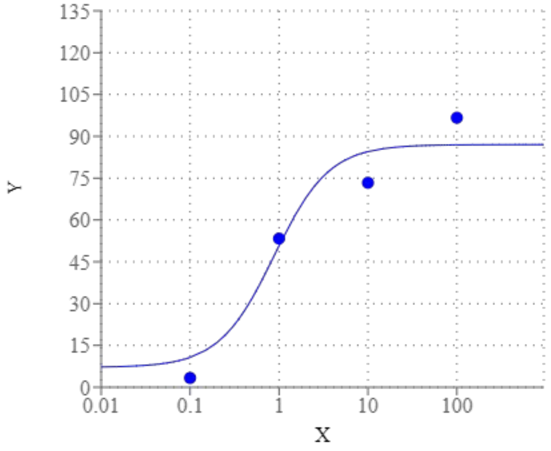	$Y = 7.1253 + \frac{87.0287 - 7.1253}{1 + (\frac{X}{0.8779})^{-1.4069}}$
	5	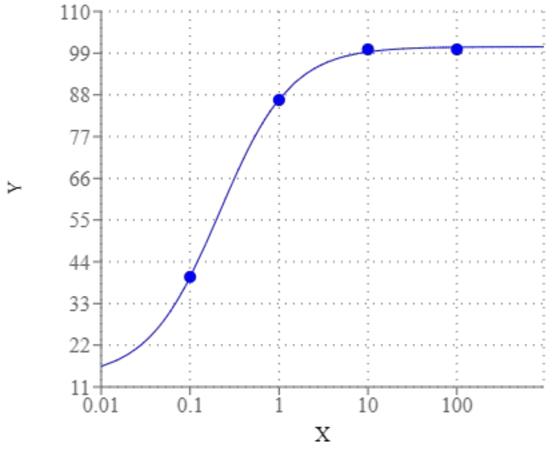	$Y = 13.3499 + \frac{100.6724 - 13.3499}{1 + (\frac{X}{0.2143})^{-1.0822}}$

<table>
<tr><td></td><td>6</td><td>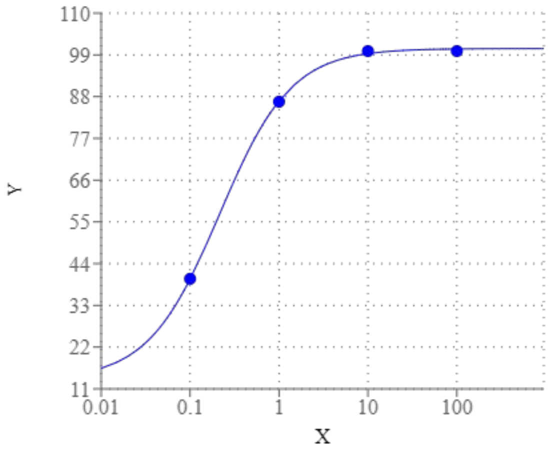
</td><td>$$Y = 13.3499 + \frac{100.6724 - 13.3499}{1 + (\frac{X}{0.2143})^{-1.0822}}$$</td></tr>
<tr><td></td><td>7</td><td>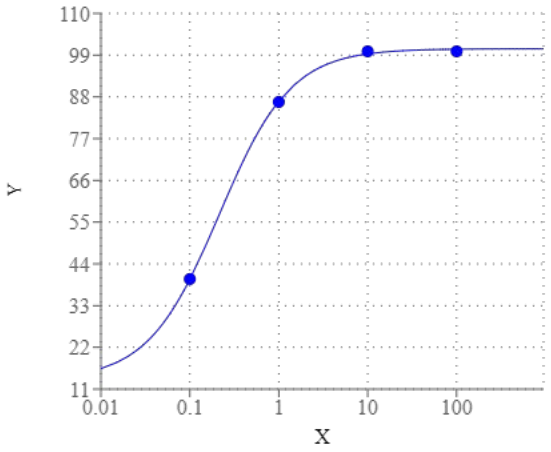
</td><td>$$Y = 13.3499 + \frac{100.6724 - 13.3499}{1 + (\frac{X}{0.2143})^{-1.0822}}$$</td></tr>
<tr><td></td><td>8</td><td>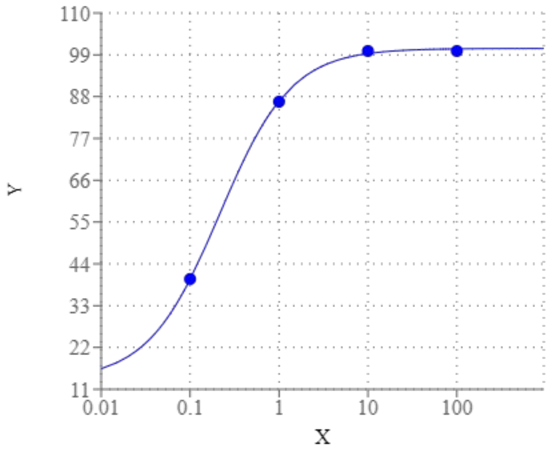
</td><td>$$Y = 13.3499 + \frac{100.6724 - 13.3499}{1 + (\frac{X}{0.2143})^{-1.0822}}$$</td></tr>
</table>

	9	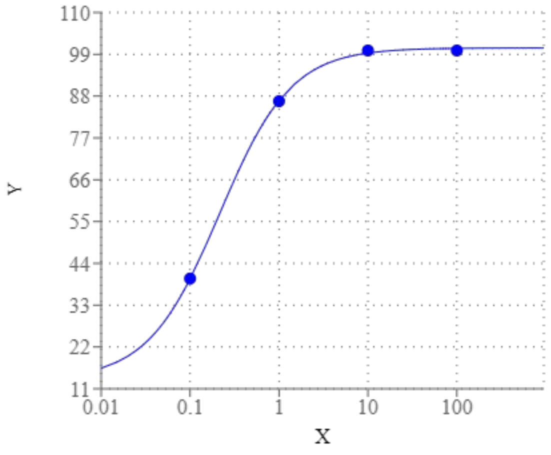	$Y = 13.3499 + \frac{100.6724 - 13.3499}{1 + (\frac{X}{0.2143})^{-1.0822}}$
	10	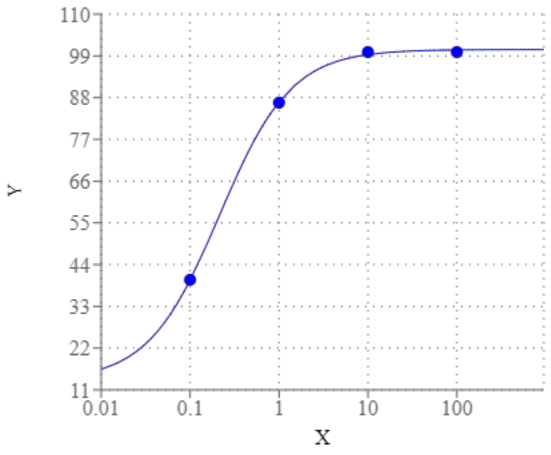	$Y = 13.3499 + \frac{100.6724 - 13.3499}{1 + (\frac{X}{0.2143})^{-1.0822}}$
	11	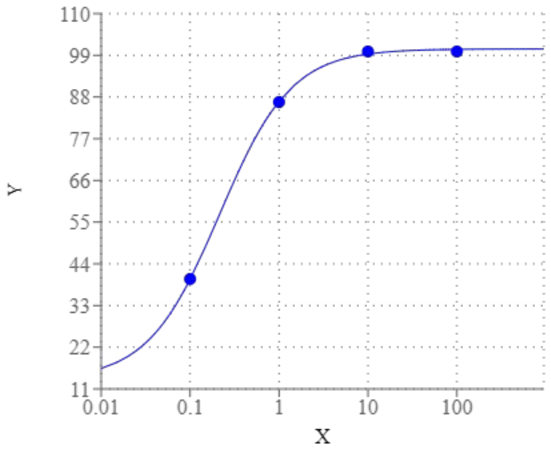	$Y = 13.3499 + \frac{100.6724 - 13.3499}{1 + (\frac{X}{0.2143})^{-1.0822}}$

Table S5. LC_{50} values for leaf-feeding mortality. 'NSE encapsulated in GNP' was quantified by normalizing the GNP LC_{50} value (Table S4) using an NSE-to-encapsulant ratio of 1:19, allowing for a lethality comparison between the contact and leaf-feeding assays.

LC_{50} [mg/mL]		**Day 2**	**Day 3**	**Day 4**	**Day 5**	**Day 6**	**Day 7**	**Day 8**	**Day 9**	**Day 10**	**Day 11**
NSE encapsulated in GNP	Contact assay	-*	-*	7.2	1.4	0.21	0.13	0.15	0.15	0.15	0.16
NSE encapsulated in GNP	Leaf-feeding assay	3.6	0.28	0.04	0.01	0.01	0.01	0.01	0.01	0.01	0.01

* When the average mortality of the highest concentration is <50%, we are unable to interpolate the LC50 value.
